# Measurement of colour flow using jet-pull observables in $t\bar{t}$ events with the ATLAS experiment at $\sqrt{s} = 13\,\hbox {TeV}$

**DOI:** 10.1140/epjc/s10052-018-6290-2

**Published:** 2018-10-22

**Authors:** M. Aaboud, G. Aad, B. Abbott, O. Abdinov, B. Abeloos, S. H. Abidi, O. S. AbouZeid, N. L. Abraham, H. Abramowicz, H. Abreu, Y. Abulaiti, B. S. Acharya, S. Adachi, L. Adamczyk, J. Adelman, M. Adersberger, T. Adye, A. A. Affolder, Y. Afik, C. Agheorghiesei, J. A. Aguilar-Saavedra, F. Ahmadov, G. Aielli, S. Akatsuka, T. P. A. Åkesson, E. Akilli, A. V. Akimov, G. L. Alberghi, J. Albert, P. Albicocco, M. J. Alconada Verzini, S. Alderweireldt, M. Aleksa, I. N. Aleksandrov, C. Alexa, G. Alexander, T. Alexopoulos, M. Alhroob, B. Ali, G. Alimonti, J. Alison, S. P. Alkire, C. Allaire, B. M. M. Allbrooke, B. W. Allen, P. P. Allport, A. Aloisio, A. Alonso, F. Alonso, C. Alpigiani, A. A. Alshehri, M. I. Alstaty, B. Alvarez Gonzalez, D. Álvarez Piqueras, M. G. Alviggi, B. T. Amadio, Y. Amaral Coutinho, C. Amelung, D. Amidei, S. P. Amor Dos Santos, S. Amoroso, C. Anastopoulos, L. S. Ancu, N. Andari, T. Andeen, C. F. Anders, J. K. Anders, K. J. Anderson, A. Andreazza, V. Andrei, S. Angelidakis, I. Angelozzi, A. Angerami, A. V. Anisenkov, A. Annovi, C. Antel, M. Antonelli, A. Antonov, D. J. A. Antrim, F. Anulli, M. Aoki, L. Aperio Bella, G. Arabidze, Y. Arai, J. P. Araque, V. Araujo Ferraz, A. T. H. Arce, R. E. Ardell, F. A. Arduh, J-F. Arguin, S. Argyropoulos, A. J. Armbruster, L. J. Armitage, O. Arnaez, H. Arnold, M. Arratia, O. Arslan, A. Artamonov, G. Artoni, S. Artz, S. Asai, N. Asbah, A. Ashkenazi, L. Asquith, K. Assamagan, R. Astalos, R. J. Atkin, M. Atkinson, N. B. Atlay, K. Augsten, G. Avolio, B. Axen, M. K. Ayoub, G. Azuelos, A. E. Baas, M. J. Baca, H. Bachacou, K. Bachas, M. Backes, P. Bagnaia, M. Bahmani, H. Bahrasemani, J. T. Baines, M. Bajic, O. K. Baker, P. J. Bakker, D. Bakshi Gupta, E. M. Baldin, P. Balek, F. Balli, W. K. Balunas, E. Banas, A. Bandyopadhyay, S. Banerjee, A. A. E. Bannoura, L. Barak, E. L. Barberio, D. Barberis, M. Barbero, T. Barillari, M-S. Barisits, J. Barkeloo, T. Barklow, N. Barlow, S. L. Barnes, B. M. Barnett, R. M. Barnett, Z. Barnovska-Blenessy, A. Baroncelli, G. Barone, A. J. Barr, L. Barranco Navarro, F. Barreiro, J. Barreiro Guimarães da Costa, R. Bartoldus, A. E. Barton, P. Bartos, A. Basalaev, A. Bassalat, R. L. Bates, S. J. Batista, J. R. Batley, M. Battaglia, M. Bauce, F. Bauer, K. T. Bauer, H. S. Bawa, J. B. Beacham, M. D. Beattie, T. Beau, P. H. Beauchemin, P. Bechtle, H. C. Beck, H. P. Beck, K. Becker, M. Becker, C. Becot, A. Beddall, A. J. Beddall, V. A. Bednyakov, M. Bedognetti, C. P. Bee, T. A. Beermann, M. Begalli, M. Begel, J. K. Behr, A. S. Bell, G. Bella, L. Bellagamba, A. Bellerive, M. Bellomo, K. Belotskiy, N. L. Belyaev, O. Benary, D. Benchekroun, M. Bender, N. Benekos, Y. Benhammou, E. Benhar Noccioli, J. Benitez, D. P. Benjamin, M. Benoit, J. R. Bensinger, S. Bentvelsen, L. Beresford, M. Beretta, D. Berge, E. Bergeaas Kuutmann, N. Berger, L. J. Bergsten, J. Beringer, S. Berlendis, N. R. Bernard, G. Bernardi, C. Bernius, F. U. Bernlochner, T. Berry, P. Berta, C. Bertella, G. Bertoli, I. A. Bertram, C. Bertsche, G. J. Besjes, O. Bessidskaia Bylund, M. Bessner, N. Besson, A. Bethani, S. Bethke, A. Betti, A. J. Bevan, J. Beyer, R. M. Bianchi, O. Biebel, D. Biedermann, R. Bielski, K. Bierwagen, N. V. Biesuz, M. Biglietti, T. R. V. Billoud, M. Bindi, A. Bingul, C. Bini, S. Biondi, T. Bisanz, C. Bittrich, D. M. Bjergaard, J. E. Black, K. M. Black, R. E. Blair, T. Blazek, I. Bloch, C. Blocker, A. Blue, U. Blumenschein, Dr. Blunier, G. J. Bobbink, V. S. Bobrovnikov, S. S. Bocchetta, A. Bocci, C. Bock, D. Boerner, D. Bogavac, A. G. Bogdanchikov, C. Bohm, V. Boisvert, P. Bokan, T. Bold, A. S. Boldyrev, A. E. Bolz, M. Bomben, M. Bona, J. S. Bonilla, M. Boonekamp, A. Borisov, G. Borissov, J. Bortfeldt, D. Bortoletto, V. Bortolotto, D. Boscherini, M. Bosman, J. D. Bossio Sola, J. Boudreau, E. V. Bouhova-Thacker, D. Boumediene, C. Bourdarios, S. K. Boutle, A. Boveia, J. Boyd, I. R. Boyko, A. J. Bozson, J. Bracinik, A. Brandt, G. Brandt, O. Brandt, F. Braren, U. Bratzler, B. Brau, J. E. Brau, W. D. Breaden Madden, K. Brendlinger, A. J. Brennan, L. Brenner, R. Brenner, S. Bressler, D. L. Briglin, T. M. Bristow, D. Britton, D. Britzger, I. Brock, R. Brock, G. Brooijmans, T. Brooks, W. K. Brooks, E. Brost, J. H Broughton, P. A. Bruckman de Renstrom, D. Bruncko, A. Bruni, G. Bruni, L. S. Bruni, S. Bruno, B. H. Brunt, M. Bruschi, N. Bruscino, P. Bryant, L. Bryngemark, T. Buanes, Q. Buat, P. Buchholz, A. G. Buckley, I. A. Budagov, M. K. Bugge, F. Bührer, O. Bulekov, D. Bullock, T. J. Burch, S. Burdin, C. D. Burgard, A. M. Burger, B. Burghgrave, K. Burka, S. Burke, I. Burmeister, J. T. P. Burr, D. Büscher, V. Büscher, E. Buschmann, P. Bussey, J. M. Butler, C. M. Buttar, J. M. Butterworth, P. Butti, W. Buttinger, A. Buzatu, A. R. Buzykaev, S. Cabrera Urbán, D. Caforio, H. Cai, V. M. M. Cairo, O. Cakir, N. Calace, P. Calafiura, A. Calandri, G. Calderini, P. Calfayan, G. Callea, L. P. Caloba, S. Calvente Lopez, D. Calvet, S. Calvet, T. P. Calvet, R. Camacho Toro, S. Camarda, P. Camarri, D. Cameron, R. Caminal Armadans, C. Camincher, S. Campana, M. Campanelli, A. Camplani, A. Campoverde, V. Canale, M. Cano Bret, J. Cantero, T. Cao, M. D. M. Capeans Garrido, I. Caprini, M. Caprini, M. Capua, R. M. Carbone, R. Cardarelli, F. C. Cardillo, I. Carli, T. Carli, G. Carlino, B. T. Carlson, L. Carminati, R. M. D. Carney, S. Caron, E. Carquin, S. Carrá, G. D. Carrillo-Montoya, D. Casadei, M. P. Casado, A. F. Casha, M. Casolino, D. W. Casper, R. Castelijn, V. Castillo Gimenez, N. F. Castro, A. Catinaccio, J. R. Catmore, A. Cattai, J. Caudron, V. Cavaliere, E. Cavallaro, D. Cavalli, M. Cavalli-Sforza, V. Cavasinni, E. Celebi, F. Ceradini, L. Cerda Alberich, A. S. Cerqueira, A. Cerri, L. Cerrito, F. Cerutti, A. Cervelli, S. A. Cetin, A. Chafaq, D Chakraborty, S. K. Chan, W. S. Chan, Y. L. Chan, P. Chang, J. D. Chapman, D. G. Charlton, C. C. Chau, C. A. Chavez Barajas, S. Che, A. Chegwidden, S. Chekanov, S. V. Chekulaev, G. A. Chelkov, M. A. Chelstowska, C. Chen, C. H. Chen, H. Chen, J. Chen, J. Chen, S. Chen, S. J. Chen, X. Chen, Y. Chen, H. C. Cheng, H. J. Cheng, A. Cheplakov, E. Cheremushkina, R. Cherkaoui El Moursli, E. Cheu, K. Cheung, L. Chevalier, V. Chiarella, G. Chiarelli, G. Chiodini, A. S. Chisholm, A. Chitan, Y. H. Chiu, M. V. Chizhov, K. Choi, A. R. Chomont, S. Chouridou, Y. S. Chow, V. Christodoulou, M. C. Chu, J. Chudoba, A. J. Chuinard, J. J. Chwastowski, L. Chytka, D. Cinca, V. Cindro, I. A. Cioară, A. Ciocio, F. Cirotto, Z. H. Citron, M. Citterio, A. Clark, M. R. Clark, P. J. Clark, R. N. Clarke, C. Clement, Y. Coadou, M. Cobal, A. Coccaro, J. Cochran, L. Colasurdo, B. Cole, A. P. Colijn, J. Collot, P. Conde Muiño, E. Coniavitis, S. H. Connell, I. A. Connelly, S. Constantinescu, G. Conti, F. Conventi, A. M. Cooper-Sarkar, F. Cormier, K. J. R. Cormier, M. Corradi, E. E. Corrigan, F. Corriveau, A. Cortes-Gonzalez, M. J. Costa, D. Costanzo, G. Cottin, G. Cowan, B. E. Cox, K. Cranmer, S. J. Crawley, R. A. Creager, G. Cree, S. Crépé-Renaudin, F. Crescioli, M. Cristinziani, V. Croft, G. Crosetti, A. Cueto, T. Cuhadar Donszelmann, A. R. Cukierman, J. Cummings, M. Curatolo, J. Cúth, S. Czekierda, P. Czodrowski, M. J. Da Cunha Sargedas De Sousa, C. Da Via, W. Dabrowski, T. Dado, S. Dahbi, T. Dai, O. Dale, F. Dallaire, C. Dallapiccola, M. Dam, G. D’amen, J. R. Dandoy, M. F. Daneri, N. P. Dang, N. D Dann, M. Danninger, M. Dano Hoffmann, V. Dao, G. Darbo, S. Darmora, J. Dassoulas, A. Dattagupta, T. Daubney, S. D’Auria, W. Davey, C. David, T. Davidek, D. R. Davis, P. Davison, E. Dawe, I. Dawson, K. De, R. De Asmundis, A. De Benedetti, S. De Castro, S. De Cecco, N. De Groot, P. de Jong, H. De la Torre, F. De Lorenzi, A. De Maria, D. De Pedis, A. De Salvo, U. De Sanctis, A. De Santo, K. De Vasconcelos Corga, J. B. De Vivie De Regie, C. Debenedetti, D. V. Dedovich, N. Dehghanian, I. Deigaard, M. Del Gaudio, J. Del Peso, D. Delgove, F. Deliot, C. M. Delitzsch, M. Della Pietra, D. Della Volpe, A. Dell’Acqua, L. Dell’Asta, M. Delmastro, C. Delporte, P. A. Delsart, D. A. DeMarco, S. Demers, M. Demichev, S. P. Denisov, D. Denysiuk, L. D’Eramo, D. Derendarz, J. E. Derkaoui, F. Derue, P. Dervan, K. Desch, C. Deterre, K. Dette, M. R. Devesa, P. O. Deviveiros, A. Dewhurst, S. Dhaliwal, F. A. Di Bello, A. Di Ciaccio, L. Di Ciaccio, W. K. Di Clemente, C. Di Donato, A. Di Girolamo, B. Di Micco, R. Di Nardo, K. F. Di Petrillo, A. Di Simone, R. Di Sipio, D. Di Valentino, C. Diaconu, M. Diamond, F. A. Dias, M. A. Diaz, J. Dickinson, E. B. Diehl, J. Dietrich, S. Díez Cornell, A. Dimitrievska, J. Dingfelder, P. Dita, S. Dita, F. Dittus, F. Djama, T. Djobava, J. I. Djuvsland, M. A. B. Do Vale, M. Dobre, D. Dodsworth, C. Doglioni, J. Dolejsi, Z. Dolezal, M. Donadelli, S. Donati, J. Donini, M. D’Onofrio, J. Dopke, A. Doria, M. T. Dova, A. T. Doyle, E. Drechsler, E. Dreyer, M. Dris, Y. Du, J. Duarte-Campderros, F. Dubinin, A. Dubreuil, E. Duchovni, G. Duckeck, A. Ducourthial, O. A. Ducu, D. Duda, A. Dudarev, A. C. Dudder, E. M. Duffield, L. Duflot, M. Dührssen, C. Dülsen, M. Dumancic, A. E. Dumitriu, A. K. Duncan, M. Dunford, A. Duperrin, H. DuranYildiz, M. Düren, A. Durglishvili, D. Duschinger, B. Dutta, D. Duvnjak, M. Dyndal, B. S. Dziedzic, C. Eckardt, K. M. Ecker, R. C. Edgar, T. Eifert, G. Eigen, K. Einsweiler, T. Ekelof, M. El Kacimi, R. El Kosseifi, V. Ellajosyula, M. Ellert, F. Ellinghaus, A. A. Elliot, N. Ellis, J. Elmsheuser, M. Elsing, D. Emeliyanov, Y. Enari, J. S. Ennis, M. B. Epland, J. Erdmann, A. Ereditato, S. Errede, M. Escalier, C. Escobar, B. Esposito, O. EstradaPastor, A. I. Etienvre, E. Etzion, H. Evans, A. Ezhilov, M. Ezzi, F. Fabbri, L. Fabbri, V. Fabiani, G. Facini, R. M. Fakhrutdinov, S. Falciano, R. J. Falla, J. Faltova, Y. Fang, M. Fanti, A. Farbin, A. Farilla, E. M. Farina, T. Farooque, S. Farrell, S. M. Farrington, P. Farthouat, F. Fassi, P. Fassnacht, D. Fassouliotis, M. Faucci Giannelli, A. Favareto, W. J. Fawcett, L. Fayard, O. L. Fedin, W. Fedorko, M. Feickert, S. Feigl, L. Feligioni, C. Feng, E. J. Feng, H. Feng, M. J. Fenton, A. B. Fenyuk, L. Feremenga, P. Fernandez Martinez, J. Ferrando, A. Ferrari, P. Ferrari, R. Ferrari, D. E. Ferreira de Lima, A. Ferrer, D. Ferrere, C. Ferretti, F. Fiedler, A. Filipčič, F. Filthaut, M. Fincke-Keeler, K. D. Finelli, M. C. N. Fiolhais, L. Fiorini, C. Fischer, J. Fischer, W. C. Fisher, N. Flaschel, I. Fleck, P. Fleischmann, R. R. M. Fletcher, T. Flick, B. M. Flierl, L. M. Flores, L. R. Flores Castillo, N. Fomin, G. T. Forcolin, A. Formica, F. A. Förster, A. C. Forti, A. G. Foster, D. Fournier, H. Fox, S. Fracchia, P. Francavilla, M. Franchini, S. Franchino, D. Francis, L. Franconi, M. Franklin, M. Frate, M. Fraternali, D. Freeborn, S. M. Fressard-Batraneanu, B. Freund, W. S. Freund, D. Froidevaux, J. A. Frost, C. Fukunaga, T. Fusayasu, J. Fuster, O. Gabizon, A. Gabrielli, A. Gabrielli, G. P. Gach, S. Gadatsch, S. Gadomski, G. Gagliardi, L. G. Gagnon, C. Galea, B. Galhardo, E. J. Gallas, B. J. Gallop, P. Gallus, G. Galster, K. K. Gan, S. Ganguly, Y. Gao, Y. S. Gao, C. García, J. E. García Navarro, J. A. García Pascual, M. Garcia-Sciveres, R. W. Gardner, N. Garelli, V. Garonne, K. Gasnikova, A. Gaudiello, G. Gaudio, I. L. Gavrilenko, C. Gay, G. Gaycken, E. N. Gazis, C. N. P. Gee, J. Geisen, M. Geisen, M. P. Geisler, K. Gellerstedt, C. Gemme, M. H. Genest, C. Geng, S. Gentile, C. Gentsos, S. George, D. Gerbaudo, G. Gessner, S. Ghasemi, M. Ghneimat, B. Giacobbe, S. Giagu, N. Giangiacomi, P. Giannetti, S. M. Gibson, M. Gignac, M. Gilchriese, D. Gillberg, G. Gilles, D. M. Gingrich, M. P. Giordani, F. M. Giorgi, P. F. Giraud, P. Giromini, G. Giugliarelli, D. Giugni, F. Giuli, M. Giulini, S. Gkaitatzis, I. Gkialas, E. L. Gkougkousis, P. Gkountoumis, L. K. Gladilin, C. Glasman, J. Glatzer, P. C. F. Glaysher, A. Glazov, M. Goblirsch-Kolb, J. Godlewski, S. Goldfarb, T. Golling, D. Golubkov, A. Gomes, R. Goncalves Gama, R. Gonçalo, G. Gonella, L. Gonella, A. Gongadze, F. Gonnella, J. L. Gonski, S. González de la Hoz, S. Gonzalez-Sevilla, L. Goossens, P. A. Gorbounov, H. A. Gordon, B. Gorini, E. Gorini, A. Gorišek, A. T. Goshaw, C. Gössling, M. I. Gostkin, C. A. Gottardo, C. R. Goudet, D. Goujdami, A. G. Goussiou, N. Govender, C. Goy, E. Gozani, I. Grabowska-Bold, P. O. J. Gradin, E. C. Graham, J. Gramling, E. Gramstad, S. Grancagnolo, V. Gratchev, P. M. Gravila, C. Gray, H. M. Gray, Z. D. Greenwood, C. Grefe, K. Gregersen, I. M. Gregor, P. Grenier, K. Grevtsov, J. Griffiths, A. A. Grillo, K. Grimm, S. Grinstein, Ph. Gris, J.-F. Grivaz, S. Groh, E. Gross, J. Grosse-Knetter, G. C. Grossi, Z. J. Grout, A. Grummer, L. Guan, W. Guan, J. Guenther, A. Guerguichon, F. Guescini, D. Guest, O. Gueta, R. Gugel, B. Gui, T. Guillemin, S. Guindon, U. Gul, C. Gumpert, J. Guo, W. Guo, Y. Guo, R. Gupta, S. Gurbuz, G. Gustavino, B. J. Gutelman, P. Gutierrez, N. G. Gutierrez Ortiz, C. Gutschow, C. Guyot, M. P. Guzik, C. Gwenlan, C. B. Gwilliam, A. Haas, C. Haber, H. K. Hadavand, N. Haddad, A. Hadef, S. Hageböck, M. Hagihara, H. Hakobyan, M. Haleem, J. Haley, G. Halladjian, G. D. Hallewell, K. Hamacher, P. Hamal, K. Hamano, A. Hamilton, G. N. Hamity, K. Han, L. Han, S. Han, K. Hanagaki, M. Hance, D. M. Handl, B. Haney, R. Hankache, P. Hanke, E. Hansen, J. B. Hansen, J. D. Hansen, M. C. Hansen, P. H. Hansen, K. Hara, A. S. Hard, T. Harenberg, F. Hariri, S. Harkusha, P. F. Harrison, N. M. Hartmann, Y. Hasegawa, A. Hasib, S. Hassani, S. Haug, R. Hauser, L. Hauswald, L. B. Havener, M. Havranek, C. M. Hawkes, R. J. Hawkings, D. Hayden, C. P. Hays, J. M. Hays, H. S. Hayward, S. J. Haywood, T. Heck, V. Hedberg, L. Heelan, S. Heer, K. K. Heidegger, S. Heim, T. Heim, B. Heinemann, J. J. Heinrich, L. Heinrich, C. Heinz, J. Hejbal, L. Helary, A. Held, S. Hellman, C. Helsens, R. C. W. Henderson, Y. Heng, S. Henkelmann, A. M. Henriques Correia, G. H. Herbert, H. Herde, V. Herget, Y. Hernández Jiménez, H. Herr, G. Herten, R. Hertenberger, L. Hervas, T. C. Herwig, G. G. Hesketh, N. P. Hessey, J. W. Hetherly, S. Higashino, E. Higón-Rodriguez, K. Hildebrand, E. Hill, J. C. Hill, K. H. Hiller, S. J. Hillier, M. Hils, I. Hinchliffe, M. Hirose, D. Hirschbuehl, B. Hiti, O. Hladik, D. R. Hlaluku, X. Hoad, J. Hobbs, N. Hod, M. C. Hodgkinson, A. Hoecker, M. R. Hoeferkamp, F. Hoenig, D. Hohn, D. Hohov, T. R. Holmes, M. Holzbock, M. Homann, S. Honda, T. Honda, T. M. Hong, B. H. Hooberman, W. H. Hopkins, Y. Horii, A. J. Horton, J-Y. Hostachy, A. Hostiuc, S. Hou, A. Hoummada, J. Howarth, J. Hoya, M. Hrabovsky, J. Hrdinka, I. Hristova, J. Hrivnac, A. Hrynevich, T. Hryn’ova, P. J. Hsu, S.-C. Hsu, Q. Hu, S. Hu, Y. Huang, Z. Hubacek, F. Hubaut, F. Huegging, T. B. Huffman, E. W. Hughes, M. Huhtinen, R. F. H. Hunter, P. Huo, A. M. Hupe, N. Huseynov, J. Huston, J. Huth, R. Hyneman, G. Iacobucci, G. Iakovidis, I. Ibragimov, L. Iconomidou-Fayard, Z. Idrissi, P. Iengo, O. Igonkina, R. Iguchi, T. Iizawa, Y. Ikegami, M. Ikeno, D. Iliadis, N. Ilic, F. Iltzsche, G. Introzzi, M. Iodice, K. Iordanidou, V. Ippolito, M. F. Isacson, N. Ishijima, M. Ishino, M. Ishitsuka, C. Issever, S. Istin, F. Ito, J. M. Iturbe Ponce, R. Iuppa, H. Iwasaki, J. M. Izen, V. Izzo, S. Jabbar, P. Jackson, R. M. Jacobs, V. Jain, G. Jäkel, K. B. Jakobi, K. Jakobs, S. Jakobsen, T. Jakoubek, D. O. Jamin, D. K. Jana, R. Jansky, J. Janssen, M. Janus, P. A. Janus, G. Jarlskog, N. Javadov, T. Javůrek, M. Javurkova, F. Jeanneau, L. Jeanty, J. Jejelava, A. Jelinskas, P. Jenni, C. Jeske, S. Jézéquel, H. Ji, J. Jia, H. Jiang, Y. Jiang, Z. Jiang, S. Jiggins, J. Jimenez Pena, S. Jin, A. Jinaru, O. Jinnouchi, H. Jivan, P. Johansson, K. A. Johns, C. A. Johnson, W. J. Johnson, K. Jon-And, R. W. L. Jones, S. D. Jones, S. Jones, T. J. Jones, J. Jongmanns, P. M. Jorge, J. Jovicevic, X. Ju, A. Juste Rozas, A. Kaczmarska, M. Kado, H. Kagan, M. Kagan, S. J. Kahn, T. Kaji, E. Kajomovitz, C. W. Kalderon, A. Kaluza, S. Kama, A. Kamenshchikov, L. Kanjir, Y. Kano, V. A. Kantserov, J. Kanzaki, B. Kaplan, L. S. Kaplan, D. Kar, K. Karakostas, N. Karastathis, M. J. Kareem, E. Karentzos, S. N. Karpov, Z. M. Karpova, V. Kartvelishvili, A. N. Karyukhin, K. Kasahara, L. Kashif, R. D. Kass, A. Kastanas, Y. Kataoka, C. Kato, A. Katre, J. Katzy, K. Kawade, K. Kawagoe, T. Kawamoto, G. Kawamura, E. F. Kay, V. F. Kazanin, R. Keeler, R. Kehoe, J. S. Keller, E. Kellermann, J. J. Kempster, J Kendrick, H. Keoshkerian, O. Kepka, S. Kersten, B. P. Kerševan, R. A. Keyes, M. Khader, F. Khalil-Zada, A. Khanov, A. G. Kharlamov, T. Kharlamova, A. Khodinov, T. J. Khoo, V. Khovanskiy, E. Khramov, J. Khubua, S. Kido, M. Kiehn, C. R. Kilby, H. Y. Kim, S. H. Kim, Y. K. Kim, N. Kimura, O. M. Kind, B. T. King, D. Kirchmeier, J. Kirk, A. E. Kiryunin, T. Kishimoto, D. Kisielewska, V. Kitali, O. Kivernyk, E. Kladiva, T. Klapdor-Kleingrothaus, M. H. Klein, M. Klein, U. Klein, K. Kleinknecht, P. Klimek, A. Klimentov, R. Klingenberg, T. Klingl, T. Klioutchnikova, F. F. Klitzner, P. Kluit, S. Kluth, E. Kneringer, E. B. F. G. Knoops, A. Knue, A. Kobayashi, D. Kobayashi, T. Kobayashi, M. Kobel, M. Kocian, P. Kodys, T. Koffas, E. Koffeman, N. M. Köhler, T. Koi, M. Kolb, I. Koletsou, T. Kondo, N. Kondrashova, K. Köneke, A. C. König, T. Kono, R. Konoplich, N. Konstantinidis, B. Konya, R. Kopeliansky, S. Koperny, K. Korcyl, K. Kordas, A. Korn, I. Korolkov, E. V. Korolkova, O. Kortner, S. Kortner, T. Kosek, V. V. Kostyukhin, A. Kotwal, A. Koulouris, A. Kourkoumeli-Charalampidi, C. Kourkoumelis, E. Kourlitis, V. Kouskoura, A. B. Kowalewska, R. Kowalewski, T. Z. Kowalski, C. Kozakai, W. Kozanecki, A. S. Kozhin, V. A. Kramarenko, G. Kramberger, D. Krasnopevtsev, M. W. Krasny, A. Krasznahorkay, D. Krauss, J. A. Kremer, J. Kretzschmar, K. Kreutzfeldt, P. Krieger, K. Krizka, K. Kroeninger, H. Kroha, J. Kroll, J. Kroll, J. Kroseberg, J. Krstic, U. Kruchonak, H. Krüger, N. Krumnack, M. C. Kruse, T. Kubota, S. Kuday, J. T. Kuechler, S. Kuehn, A. Kugel, F. Kuger, T. Kuhl, V. Kukhtin, R. Kukla, Y. Kulchitsky, S. Kuleshov, Y. P. Kulinich, M. Kuna, T. Kunigo, A. Kupco, T. Kupfer, O. Kuprash, H. Kurashige, L. L. Kurchaninov, Y. A. Kurochkin, M. G. Kurth, E. S. Kuwertz, M. Kuze, J. Kvita, T. Kwan, A. La Rosa, J. L. La Rosa Navarro, L. La Rotonda, F. La Ruffa, C. Lacasta, F. Lacava, J. Lacey, D. P. J. Lack, H. Lacker, D. Lacour, E. Ladygin, R. Lafaye, B. Laforge, S. Lai, S. Lammers, W. Lampl, E. Lançon, U. Landgraf, M. P. J. Landon, M. C. Lanfermann, V. S. Lang, J. C. Lange, R. J. Langenberg, A. J. Lankford, F. Lanni, K. Lantzsch, A. Lanza, A. Lapertosa, S. Laplace, J. F. Laporte, T. Lari, F. Lasagni Manghi, M. Lassnig, T. S. Lau, A. Laudrain, A. T. Law, P. Laycock, M. Lazzaroni, B. Le, O. Le Dortz, E. Le Guirriec, E. P. Le Quilleuc, M. LeBlanc, T. LeCompte, F. Ledroit-Guillon, C. A. Lee, G. R. Lee, L. Lee, S. C. Lee, B. Lefebvre, M. Lefebvre, F. Legger, C. Leggett, G. Lehmann Miotto, X. Lei, W. A. Leight, A. Leisos, M. A. L. Leite, R. Leitner, D. Lellouch, B. Lemmer, K. J. C. Leney, T. Lenz, B. Lenzi, R. Leone, S. Leone, C. Leonidopoulos, G. Lerner, C. Leroy, R. Les, A. A. J. Lesage, C. G. Lester, M. Levchenko, J. Levêque, D. Levin, L. J. Levinson, M. Levy, D. Lewis, B. Li, C-Q. Li, H. Li, L. Li, Q. Li, Q. Y. Li, S. Li, X. Li, Y. Li, Z. Liang, B. Liberti, A. Liblong, K. Lie, A. Limosani, C. Y. Lin, K. Lin, S. C. Lin, T. H. Lin, R. A. Linck, B. E. Lindquist, A. L. Lionti, E. Lipeles, A. Lipniacka, M. Lisovyi, T. M. Liss, A. Lister, A. M. Litke, B. Liu, H. B. Liu, H. Liu, J. B. Liu, J. K. K. Liu, K. Liu, M. Liu, P. Liu, Y. L. Liu, Y. W. Liu, M. Livan, A. Lleres, J. Llorente Merino, S. L. Lloyd, C. Y. Lo, F. Lo Sterzo, E. M. Lobodzinska, P. Loch, F. K. Loebinger, K. M. Loew, T. Lohse, K. Lohwasser, M. Lokajicek, B. A. Long, J. D. Long, R. E. Long, L. Longo, K. A. Looper, J. A. Lopez, I. Lopez Paz, A. Lopez Solis, J. Lorenz, N. Lorenzo Martinez, M. Losada, P. J. Lösel, A. Lösle, X. Lou, A. Lounis, J. Love, P. A. Love, H. Lu, N. Lu, Y. J. Lu, H. J. Lubatti, C. Luci, A. Lucotte, C. Luedtke, F. Luehring, W. Lukas, L. Luminari, B. Lund-Jensen, M. S. Lutz, P. M. Luzi, D. Lynn, R. Lysak, E. Lytken, F. Lyu, V. Lyubushkin, H. Ma, L. L. Ma, Y. Ma, G. Maccarrone, A. Macchiolo, C. M. Macdonald, J. Machado Miguens, D. Madaffari, R. Madar, W. F. Mader, A. Madsen, N. Madysa, J. Maeda, S. Maeland, T. Maeno, A. S. Maevskiy, V. Magerl, C. Maidantchik, T. Maier, A. Maio, O. Majersky, S. Majewski, Y. Makida, N. Makovec, B. Malaescu, Pa. Malecki, V. P. Maleev, F. Malek, U. Mallik, D. Malon, C. Malone, S. Maltezos, S. Malyukov, J. Mamuzic, G. Mancini, I. Mandić, J. Maneira, L. Manhaes de Andrade Filho, J. Manjarres Ramos, K. H. Mankinen, A. Mann, A. Manousos, B. Mansoulie, J. D. Mansour, R. Mantifel, M. Mantoani, S. Manzoni, G. Marceca, L. March, L. Marchese, G. Marchiori, M. Marcisovsky, C. A. Marin Tobon, M. Marjanovic, D. E. Marley, F. Marroquim, Z. Marshall, M. U. F Martensson, S. Marti-Garcia, C. B. Martin, T. A. Martin, V. J. Martin, B. Martin dit Latour, M. Martinez, V. I. Martinez Outschoorn, S. Martin-Haugh, V. S. Martoiu, A. C. Martyniuk, A. Marzin, L. Masetti, T. Mashimo, R. Mashinistov, J. Masik, A. L. Maslennikov, L. H. Mason, L. Massa, P. Mastrandrea, A. Mastroberardino, T. Masubuchi, P. Mättig, J. Maurer, B. Maček, S. J. Maxfield, D. A. Maximov, R. Mazini, I. Maznas, S. M. Mazza, N. C. Mc Fadden, G. Mc Goldrick, S. P. Mc Kee, A. McCarn, T. G. McCarthy, L. I. McClymont, E. F. McDonald, J. A. Mcfayden, G. Mchedlidze, S. J. McMahon, P. C. McNamara, C. J. McNicol, R. A. McPherson, Z. A. Meadows, S. Meehan, T. M. Megy, S. Mehlhase, A. Mehta, T. Meideck, B. Meirose, D. Melini, B. R. Mellado Garcia, J. D. Mellenthin, M. Melo, F. Meloni, A. Melzer, S. B. Menary, L. Meng, X. T. Meng, A. Mengarelli, S. Menke, E. Meoni, S. Mergelmeyer, C. Merlassino, P. Mermod, L. Merola, C. Meroni, F. S. Merritt, A. Messina, J. Metcalfe, A. S. Mete, C. Meyer, J. Meyer, J-P. Meyer, H. Meyer Zu Theenhausen, F. Miano, R. P. Middleton, S. Miglioranzi, L. Mijović, G. Mikenberg, M. Mikestikova, M. Mikuž, M. Milesi, A. Milic, D. A. Millar, D. W. Miller, A. Milov, D. A. Milstead, A. A. Minaenko, I. A. Minashvili, A. I. Mincer, B. Mindur, M. Mineev, Y. Minegishi, Y. Ming, L. M. Mir, A. Mirto, K. P. Mistry, T. Mitani, J. Mitrevski, V. A. Mitsou, A. Miucci, P. S. Miyagawa, A. Mizukami, J. U. Mjörnmark, T. Mkrtchyan, M. Mlynarikova, T. Moa, K. Mochizuki, P. Mogg, S. Mohapatra, S. Molander, R. Moles-Valls, M. C. Mondragon, K. Mönig, J. Monk, E. Monnier, A. Montalbano, J. Montejo Berlingen, F. Monticelli, S. Monzani, R. W. Moore, N. Morange, D. Moreno, M. Moreno Llácer, P. Morettini, M. Morgenstern, S. Morgenstern, D. Mori, T. Mori, M. Morii, M. Morinaga, V. Morisbak, A. K. Morley, G. Mornacchi, J. D. Morris, L. Morvaj, P. Moschovakos, M. Mosidze, H. J. Moss, J. Moss, K. Motohashi, R. Mount, E. Mountricha, E. J. W. Moyse, S. Muanza, F. Mueller, J. Mueller, R. S. P. Mueller, D. Muenstermann, P. Mullen, G. A. Mullier, F. J. Munoz Sanchez, P. Murin, W. J. Murray, M. Muškinja, C. Mwewa, A. G. Myagkov, J. Myers, M. Myska, B. P. Nachman, O. Nackenhorst, K. Nagai, R. Nagai, K. Nagano, Y. Nagasaka, K. Nagata, M. Nagel, E. Nagy, A. M. Nairz, Y. Nakahama, K. Nakamura, T. Nakamura, I. Nakano, R. F. Naranjo Garcia, R. Narayan, D. I. Narrias Villar, I. Naryshkin, T. Naumann, G. Navarro, R. Nayyar, H. A. Neal, P. Y. Nechaeva, T. J. Neep, A. Negri, M. Negrini, S. Nektarijevic, C. Nellist, M. E. Nelson, S. Nemecek, P. Nemethy, M. Nessi, M. S. Neubauer, M. Neumann, P. R. Newman, T. Y. Ng, Y. S. Ng, T. Nguyen Manh, R. B. Nickerson, R. Nicolaidou, J. Nielsen, N. Nikiforou, V. Nikolaenko, I. Nikolic-Audit, K. Nikolopoulos, P. Nilsson, Y. Ninomiya, A. Nisati, N. Nishu, R. Nisius, I. Nitsche, T. Nitta, T. Nobe, Y. Noguchi, M. Nomachi, I. Nomidis, M. A. Nomura, T. Nooney, M. Nordberg, N. Norjoharuddeen, O. Novgorodova, R. Novotny, M. Nozaki, L. Nozka, K. Ntekas, E. Nurse, F. Nuti, F. G. Oakham, H. Oberlack, T. Obermann, J. Ocariz, A. Ochi, I. Ochoa, J. P. Ochoa-Ricoux, K. O’Connor, S. Oda, S. Odaka, A. Oh, S. H. Oh, C. C. Ohm, H. Ohman, H. Oide, H. Okawa, Y. Okumura, T. Okuyama, A. Olariu, L. F. Oleiro Seabra, S. A. Olivares Pino, D. Oliveira Damazio, J. L. Oliver, M. J. R. Olsson, A. Olszewski, J. Olszowska, D. C. O’Neil, A. Onofre, K. Onogi, P. U. E. Onyisi, H. Oppen, M. J. Oreglia, Y. Oren, D. Orestano, E. C. Orgill, N. Orlando, A. A. O’Rourke, R. S. Orr, B. Osculati, V. O’Shea, R. Ospanov, G. Otero y Garzon, H. Otono, M. Ouchrif, F. Ould-Saada, A. Ouraou, K. P. Oussoren, Q. Ouyang, M. Owen, R. E. Owen, V. E. Ozcan, N. Ozturk, K. Pachal, A. Pacheco Pages, L. Pacheco Rodriguez, C. Padilla Aranda, S. Pagan Griso, M. Paganini, F. Paige, G. Palacino, S. Palazzo, S. Palestini, M. Palka, D. Pallin, E. St. Panagiotopoulou, I. Panagoulias, C. E. Pandini, J. G. Panduro Vazquez, P. Pani, D. Pantea, L. Paolozzi, T. D. Papadopoulou, K. Papageorgiou, A. Paramonov, D. Paredes Hernandez, B. Parida, A. J. Parker, K. A. Parker, M. A. Parker, F. Parodi, J. A. Parsons, U. Parzefall, V. R. Pascuzzi, J. M. P. Pasner, E. Pasqualucci, S. Passaggio, F. Pastore, S. Pataraia, J. R. Pater, T. Pauly, B. Pearson, S. Pedraza Lopez, R. Pedro, S. V. Peleganchuk, O. Penc, C. Peng, H. Peng, J. Penwell, B. S. Peralva, M. M. Perego, D. V. Perepelitsa, F. Peri, L. Perini, H. Pernegger, S. Perrella, V. D. Peshekhonov, K. Peters, R. F. Y. Peters, B. A. Petersen, T. C. Petersen, E. Petit, A. Petridis, C. Petridou, P. Petroff, E. Petrolo, M. Petrov, F. Petrucci, N. E. Pettersson, A. Peyaud, R. Pezoa, T. Pham, F. H. Phillips, P. W. Phillips, G. Piacquadio, E. Pianori, A. Picazio, M. A. Pickering, R. Piegaia, J. E. Pilcher, A. D. Pilkington, M. Pinamonti, J. L. Pinfold, M. Pitt, M.-A. Pleier, V. Pleskot, E. Plotnikova, D. Pluth, P. Podberezko, R. Poettgen, R. Poggi, L. Poggioli, I. Pogrebnyak, D. Pohl, I. Pokharel, G. Polesello, A. Poley, A. Policicchio, R. Polifka, A. Polini, C. S. Pollard, V. Polychronakos, D. Ponomarenko, L. Pontecorvo, G. A. Popeneciu, D. M. Portillo Quintero, S. Pospisil, K. Potamianos, I. N. Potrap, C. J. Potter, H. Potti, T. Poulsen, J. Poveda, M. E. Pozo Astigarraga, P. Pralavorio, S. Prell, D. Price, M. Primavera, S. Prince, N. Proklova, K. Prokofiev, F. Prokoshin, S. Protopopescu, J. Proudfoot, M. Przybycien, A. Puri, P. Puzo, J. Qian, Y. Qin, A. Quadt, M. Queitsch-Maitland, A. Qureshi, V. Radeka, S. K. Radhakrishnan, P. Rados, F. Ragusa, G. Rahal, J. A. Raine, S. Rajagopalan, T. Rashid, S. Raspopov, M. G. Ratti, D. M. Rauch, F. Rauscher, S. Rave, I. Ravinovich, J. H. Rawling, M. Raymond, A. L. Read, N. P. Readioff, M. Reale, D. M. Rebuzzi, A. Redelbach, G. Redlinger, R. Reece, R. G. Reed, K. Reeves, L. Rehnisch, J. Reichert, A. Reiss, C. Rembser, H. Ren, M. Rescigno, S. Resconi, E. D. Resseguie, S. Rettie, E. Reynolds, O. L. Rezanova, P. Reznicek, R. Richter, S. Richter, E. Richter-Was, O. Ricken, M. Ridel, P. Rieck, C. J. Riegel, O. Rifki, M. Rijssenbeek, A. Rimoldi, M. Rimoldi, L. Rinaldi, G. Ripellino, B. Ristić, E. Ritsch, I. Riu, J. C. Rivera Vergara, F. Rizatdinova, E. Rizvi, C. Rizzi, R. T. Roberts, S. H. Robertson, A. Robichaud-Veronneau, D. Robinson, J. E. M. Robinson, A. Robson, E. Rocco, C. Roda, Y. Rodina, S. Rodriguez Bosca, A. Rodriguez Perez, D. Rodriguez Rodriguez, A. M. Rodríguez Vera, S. Roe, C. S. Rogan, O. Røhne, R. Röhrig, J. Roloff, A. Romaniouk, M. Romano, S. M. Romano Saez, E. Romero Adam, N. Rompotis, M. Ronzani, L. Roos, S. Rosati, K. Rosbach, P. Rose, N-A. Rosien, E. Rossi, L. P. Rossi, J. H. N. Rosten, R. Rosten, M. Rotaru, J. Rothberg, D. Rousseau, D. Roy, A. Rozanov, Y. Rozen, X. Ruan, F. Rubbo, F. Rühr, A. Ruiz-Martinez, Z. Rurikova, N. A. Rusakovich, H. L. Russell, J. P. Rutherfoord, N. Ruthmann, E. M. Rüttinger, Y. F. Ryabov, M. Rybar, G. Rybkin, S. Ryu, A. Ryzhov, G. F. Rzehorz, G. Sabato, S. Sacerdoti, H. F-W. Sadrozinski, R. Sadykov, F. Safai Tehrani, P. Saha, M. Sahinsoy, M. Saimpert, M. Saito, T. Saito, H. Sakamoto, G. Salamanna, J. E. Salazar Loyola, D. Salek, P. H. Sales De Bruin, D. Salihagic, A. Salnikov, J. Salt, D. Salvatore, F. Salvatore, A. Salvucci, A. Salzburger, D. Sammel, D. Sampsonidis, D. Sampsonidou, J. Sánchez, A. Sanchez Pineda, H. Sandaker, R. L. Sandbach, C. O. Sander, M. Sandhoff, C. Sandoval, D. P. C. Sankey, M. Sannino, Y. Sano, A. Sansoni, C. Santoni, H. Santos, I. Santoyo Castillo, A. Sapronov, J. G. Saraiva, O. Sasaki, K. Sato, E. Sauvan, P. Savard, N. Savic, R. Sawada, C. Sawyer, L. Sawyer, C. Sbarra, A. Sbrizzi, T. Scanlon, D. A. Scannicchio, J. Schaarschmidt, P. Schacht, B. M. Schachtner, D. Schaefer, L. Schaefer, J. Schaeffer, S. Schaepe, U. Schäfer, A. C. Schaffer, D. Schaile, R. D. Schamberger, V. A. Schegelsky, D. Scheirich, F. Schenck, M. Schernau, C. Schiavi, S. Schier, L. K. Schildgen, C. Schillo, E. J. Schioppa, M. Schioppa, K. E. Schleicher, S. Schlenker, K. R. Schmidt-Sommerfeld, K. Schmieden, C. Schmitt, S. Schmitt, S. Schmitz, U. Schnoor, L. Schoeffel, A. Schoening, E. Schopf, M. Schott, J. F. P. Schouwenberg, J. Schovancova, S. Schramm, N. Schuh, A. Schulte, H-C. Schultz-Coulon, M. Schumacher, B. A. Schumm, Ph. Schune, A. Schwartzman, T. A. Schwarz, H. Schweiger, Ph. Schwemling, R. Schwienhorst, A. Sciandra, G. Sciolla, M. Scornajenghi, F. Scuri, F. Scutti, L. M. Scyboz, J. Searcy, P. Seema, S. C. Seidel, A. Seiden, J. M. Seixas, G. Sekhniaidze, K. Sekhon, S. J. Sekula, N. Semprini-Cesari, S. Senkin, C. Serfon, L. Serin, L. Serkin, M. Sessa, H. Severini, F. Sforza, A. Sfyrla, E. Shabalina, J. D. Shahinian, N. W. Shaikh, L. Y. Shan, R. Shang, J. T. Shank, M. Shapiro, P. B. Shatalov, K. Shaw, S. M. Shaw, A. Shcherbakova, C. Y. Shehu, Y. Shen, N. Sherafati, A. D. Sherman, P. Sherwood, L. Shi, S. Shimizu, C. O. Shimmin, M. Shimojima, I. P. J. Shipsey, S. Shirabe, M. Shiyakova, J. Shlomi, A. Shmeleva, D. Shoaleh Saadi, M. J. Shochet, S. Shojaii, D. R. Shope, S. Shrestha, E. Shulga, P. Sicho, A. M. Sickles, P. E. Sidebo, E. Sideras Haddad, O. Sidiropoulou, A. Sidoti, F. Siegert, Dj. Sijacki, J. Silva, M. Silva, S. B. Silverstein, L. Simic, S. Simion, E. Simioni, B. Simmons, M. Simon, P. Sinervo, N. B. Sinev, M. Sioli, G. Siragusa, I. Siral, S. Yu. Sivoklokov, J. Sjölin, M. B. Skinner, P. Skubic, M. Slater, T. Slavicek, M. Slawinska, K. Sliwa, R. Slovak, V. Smakhtin, B. H. Smart, J. Smiesko, N. Smirnov, S. Yu. Smirnov, Y. Smirnov, L. N. Smirnova, O. Smirnova, J. W. Smith, M. N. K. Smith, R. W. Smith, M. Smizanska, K. Smolek, A. A. Snesarev, I. M. Snyder, S. Snyder, R. Sobie, F. Socher, A. M. Soffa, A. Soffer, A. Søgaard, D. A. Soh, G. Sokhrannyi, C. A. Solans Sanchez, M. Solar, E. Yu. Soldatov, U. Soldevila, A. A. Solodkov, A. Soloshenko, O. V. Solovyanov, V. Solovyev, P. Sommer, H. Son, W. Song, A. Sopczak, F. Sopkova, D. Sosa, C. L. Sotiropoulou, S. Sottocornola, R. Soualah, A. M. Soukharev, D. South, B. C. Sowden, S. Spagnolo, M. Spalla, M. Spangenberg, F. Spanò, D. Sperlich, F. Spettel, T. M. Spieker, R. Spighi, G. Spigo, L. A. Spiller, M. Spousta, R. D. St. Denis, A. Stabile, R. Stamen, S. Stamm, E. Stanecka, R. W. Stanek, C. Stanescu, M. M. Stanitzki, B. Stapf, S. Stapnes, E. A. Starchenko, G. H. Stark, J. Stark, S. H Stark, P. Staroba, P. Starovoitov, S. Stärz, R. Staszewski, M. Stegler, P. Steinberg, B. Stelzer, H. J. Stelzer, O. Stelzer-Chilton, H. Stenzel, T. J. Stevenson, G. A. Stewart, M. C. Stockton, G. Stoicea, P. Stolte, S. Stonjek, A. Straessner, M. E. Stramaglia, J. Strandberg, S. Strandberg, M. Strauss, P. Strizenec, R. Ströhmer, D. M. Strom, R. Stroynowski, A. Strubig, S. A. Stucci, B. Stugu, N. A. Styles, D. Su, J. Su, S. Suchek, Y. Sugaya, M. Suk, V. V. Sulin, D. M. S. Sultan, S. Sultansoy, T. Sumida, S. Sun, X. Sun, K. Suruliz, C. J. E. Suster, M. R. Sutton, S. Suzuki, M. Svatos, M. Swiatlowski, S. P. Swift, A. Sydorenko, I. Sykora, T. Sykora, D. Ta, K. Tackmann, J. Taenzer, A. Taffard, R. Tafirout, E. Tahirovic, N. Taiblum, H. Takai, R. Takashima, E. H. Takasugi, K. Takeda, T. Takeshita, Y. Takubo, M. Talby, A. A. Talyshev, J. Tanaka, M. Tanaka, R. Tanaka, R. Tanioka, B. B. Tannenwald, S. Tapia Araya, S. Tapprogge, A. Tarek Abouelfadl Mohamed, S. Tarem, G. Tarna, G. F. Tartarelli, P. Tas, M. Tasevsky, T. Tashiro, E. Tassi, A. Tavares Delgado, Y. Tayalati, A. C. Taylor, A. J. Taylor, G. N. Taylor, P. T. E. Taylor, W. Taylor, P. Teixeira-Dias, D. Temple, H. Ten Kate, P. K. Teng, J. J. Teoh, F. Tepel, S. Terada, K. Terashi, J. Terron, S. Terzo, M. Testa, R. J. Teuscher, S. J. Thais, T. Theveneaux-Pelzer, F. Thiele, J. P. Thomas, J. Thomas-Wilsker, A. S. Thompson, P. D. Thompson, L. A. Thomsen, E. Thomson, Y. Tian, R. E. Ticse Torres, V. O. Tikhomirov, Yu. A. Tikhonov, S. Timoshenko, P. Tipton, S. Tisserant, K. Todome, S. Todorova-Nova, S. Todt, J. Tojo, S. Tokár, K. Tokushuku, E. Tolley, M. Tomoto, L. Tompkins, K. Toms, B. Tong, P. Tornambe, E. Torrence, H. Torres, E. Torró Pastor, J. Toth, F. Touchard, D. R. Tovey, C. J. Treado, T. Trefzger, F. Tresoldi, A. Tricoli, I. M. Trigger, S. Trincaz-Duvoid, M. F. Tripiana, W. Trischuk, B. Trocmé, A. Trofymov, C. Troncon, M. Trovatelli, L. Truong, M. Trzebinski, A. Trzupek, K. W. Tsang, J. C-L. Tseng, P. V. Tsiareshka, N. Tsirintanis, S. Tsiskaridze, V. Tsiskaridze, E. G. Tskhadadze, I. I. Tsukerman, V. Tsulaia, S. Tsuno, D. Tsybychev, Y. Tu, A. Tudorache, V. Tudorache, T. T. Tulbure, A. N. Tuna, S. Turchikhin, D. Turgeman, I. Turk Cakir, R. Turra, P. M. Tuts, G. Ucchielli, I. Ueda, M. Ughetto, F. Ukegawa, G. Unal, A. Undrus, G. Unel, F. C. Ungaro, Y. Unno, K. Uno, J. Urban, P. Urquijo, P. Urrejola, G. Usai, J. Usui, L. Vacavant, V. Vacek, B. Vachon, K. O. H. Vadla, A. Vaidya, C. Valderanis, E. Valdes Santurio, M. Valente, S. Valentinetti, A. Valero, L. Valéry, A. Vallier, J. A. Valls Ferrer, W. Van Den Wollenberg, H. Van der Graaf, P. Van Gemmeren, J. Van Nieuwkoop, I. Van Vulpen, M. C. van Woerden, M. Vanadia, W. Vandelli, A. Vaniachine, P. Vankov, R. Vari, E. W. Varnes, C. Varni, T. Varol, D. Varouchas, A. Vartapetian, K. E. Varvell, G. A. Vasquez, J. G. Vasquez, F. Vazeille, D. Vazquez Furelos, T. Vazquez Schroeder, J. Veatch, L. M. Veloce, F. Veloso, S. Veneziano, A. Ventura, M. Venturi, N. Venturi, V. Vercesi, M. Verducci, W. Verkerke, A. T. Vermeulen, J. C. Vermeulen, M. C. Vetterli, N. Viaux Maira, O. Viazlo, I. Vichou, T. Vickey, O. E. Vickey Boeriu, G. H. A. Viehhauser, S. Viel, L. Vigani, M. Villa, M. Villaplana Perez, E. Vilucchi, M. G. Vincter, V. B. Vinogradov, A. Vishwakarma, C. Vittori, I. Vivarelli, S. Vlachos, M. Vogel, P. Vokac, G. Volpi, S. E. von Buddenbrock, E. Von Toerne, V. Vorobel, K. Vorobev, M. Vos, J. H. Vossebeld, N. Vranjes, M. Vranjes Milosavljevic, V. Vrba, M. Vreeswijk, T. Šfiligoj, R. Vuillermet, I. Vukotic, T. Ženiš, L. Živković, P. Wagner, W. Wagner, J. Wagner-Kuhr, H. Wahlberg, S. Wahrmund, K. Wakamiya, J. Walder, R. Walker, W. Walkowiak, V. Wallangen, A. M. Wang, C. Wang, F. Wang, H. Wang, H. Wang, J. Wang, J. Wang, Q. Wang, R. -J. Wang, R. Wang, S. M. Wang, T. Wang, W. Wang, W. X. Wang, Z. Wang, C. Wanotayaroj, A. Warburton, C. P. Ward, D. R. Wardrope, A. Washbrook, P. M. Watkins, A. T. Watson, M. F. Watson, G. Watts, S. Watts, B. M. Waugh, A. F. Webb, S. Webb, M. S. Weber, S. A. Weber, S. M. Weber, J. S. Webster, A. R. Weidberg, B. Weinert, J. Weingarten, M. Weirich, C. Weiser, P. S. Wells, T. Wenaus, T. Wengler, S. Wenig, N. Wermes, M. D. Werner, P. Werner, M. Wessels, T. D. Weston, K. Whalen, N. L. Whallon, A. M. Wharton, A. S. White, A. White, M. J. White, R. White, D. Whiteson, B. W. Whitmore, F. J. Wickens, W. Wiedenmann, M. Wielers, C. Wiglesworth, L. A. M. Wiik-Fuchs, A. Wildauer, F. Wilk, H. G. Wilkens, H. H. Williams, S. Williams, C. Willis, S. Willocq, J. A. Wilson, I. Wingerter-Seez, E. Winkels, F. Winklmeier, O. J. Winston, B. T. Winter, M. Wittgen, M. Wobisch, A. Wolf, T. M. H. Wolf, R. Wolff, M. W. Wolter, H. Wolters, V. W. S. Wong, N. L. Woods, S. D. Worm, B. K. Wosiek, K. W. Woźniak, M. Wu, S. L. Wu, X. Wu, Y. Wu, T. R. Wyatt, B. M. Wynne, S. Xella, Z. Xi, L. Xia, D. Xu, L. Xu, T. Xu, W. Xu, B. Yabsley, S. Yacoob, K. Yajima, D. P. Yallup, D. Yamaguchi, Y. Yamaguchi, A. Yamamoto, T. Yamanaka, F. Yamane, M. Yamatani, T. Yamazaki, Y. Yamazaki, Z. Yan, H. J. Yang, H. T. Yang, S. Yang, Y. Yang, Z. Yang, W-M. Yao, Y. C. Yap, Y. Yasu, E. Yatsenko, K. H. Yau Wong, J. Ye, S. Ye, I. Yeletskikh, E. Yigitbasi, E. Yildirim, K. Yorita, K. Yoshihara, C. J. S. Young, C. Young, J. Yu, J. Yu, S. P. Y. Yuen, I. Yusuff, B. Zabinski, G. Zacharis, R. Zaidan, A. M. Zaitsev, N. Zakharchuk, J. Zalieckas, A. Zaman, S. Zambito, D. Zanzi, C. Zeitnitz, G. Zemaityte, J. C. Zeng, Q. Zeng, O. Zenin, D. Zerwas, D. F. Zhang, D. Zhang, F. Zhang, G. Zhang, H. Zhang, J. Zhang, L. Zhang, L. Zhang, M. Zhang, P. Zhang, R. Zhang, R. Zhang, X. Zhang, Y. Zhang, Z. Zhang, X. Zhao, Y. Zhao, Z. Zhao, A. Zhemchugov, B. Zhou, C. Zhou, L. Zhou, M. S. Zhou, M. Zhou, N. Zhou, Y. Zhou, C. G. Zhu, H. Zhu, J. Zhu, Y. Zhu, X. Zhuang, K. Zhukov, V. Zhulanov, A. Zibell, D. Zieminska, N. I. Zimine, S. Zimmermann, Z. Zinonos, M. Zinser, M. Ziolkowski, G. Zobernig, A. Zoccoli, R. Zou, M. Zur Nedden, L. Zwalinski, ATLAS Collaboration

**Affiliations:** 1 Department of Physics, University of Adelaide, Adelaide, Australia; 2 Physics Department, SUNY Albany, Albany, NY USA; 3 Department of Physics, University of Alberta, Edmonton, AB Canada; 4 Department of Physics, Ankara University, Ankara, Turkey; 5 Istanbul Aydin University, Istanbul, Turkey; 6 Division of Physics, TOBB University of Economics and Technology, Ankara, Turkey; 7 LAPP, Université Grenoble Alpes, Université Savoie Mont Blanc, CNRS/IN2P3, Annecy, France; 8 High Energy Physics Division, Argonne National Laboratory, Argonne, IL USA; 9 Department of Physics, University of Arizona, Tucson, AZ USA; 10 Department of Physics, University of Texas at Arlington, Arlington, TX USA; 11 Physics Department, National and Kapodistrian University of Athens, Athens, Greece; 12 Physics Department, National Technical University of Athens, Zografou, Greece; 13 Department of Physics, University of Texas at Austin, Austin, TX USA; 14 Bahcesehir University, Faculty of Engineering and Natural Sciences, Istanbul, Turkey; 15 Istanbul Bilgi University, Faculty of Engineering and Natural Sciences, Istanbul, Turkey; 16 Department of Physics, Bogazici University, Istanbul, Turkey; 17 Department of Physics Engineering, Gaziantep University, Gaziantep, Turkey; 18 Institute of Physics, Azerbaijan Academy of Sciences, Baku, Azerbaijan; 19 Institut de Física d’Altes Energies (IFAE), Barcelona Institute of Science and Technology, Barcelona, Spain; 20 Institute of High Energy Physics, Chinese Academy of Sciences, Beijing, China; 21 Physics Department, Tsinghua University, Beijing, China; 22 Department of Physics, Nanjing University, Nanjing, China; 23 University of Chinese Academy of Science (UCAS), Beijing, China; 24 Institute of Physics, University of Belgrade, Belgrade, Serbia; 25 Department for Physics and Technology, University of Bergen, Bergen, Norway; 26 Physics Division, Lawrence Berkeley National Laboratory and University of California, Berkeley, CA USA; 27 Institut für Physik, Humboldt Universität zu Berlin, Berlin, Germany; 28 Albert Einstein Center for Fundamental Physics and Laboratory for High Energy Physics, University of Bern, Bern, Switzerland; 29 School of Physics and Astronomy, University of Birmingham, Birmingham, UK; 30 Centro de Investigaciónes, Universidad Antonio Nariño, Bogota, Colombia; 31 Dipartimento di Fisica e Astronomia, Università di Bologna, Bologna, Italy; 32 INFN Sezione di Bologna, Bologna, Italy; 33 Physikalisches Institut, Universität Bonn, Bonn, Germany; 34 Department of Physics, Boston University, Boston, MA USA; 35 Department of Physics, Brandeis University, Waltham, MA USA; 36 Transilvania University of Brasov, Brasov, Romania; 37 Horia Hulubei National Institute of Physics and Nuclear Engineering, Bucharest, Romania; 38 Department of Physics, Alexandru Ioan Cuza University of Iasi, Iasi, Romania; 39 Physics Department, National Institute for Research and Development of Isotopic and Molecular Technologies, Cluj-Napoca, Romania; 40 University Politehnica Bucharest, Bucharest, Romania; 41 West University in Timisoara, Timisoara, Romania; 42 Faculty of Mathematics, Physics and Informatics, Comenius University, Bratislava, Slovakia; 43 Department of Subnuclear Physics, Institute of Experimental Physics of the Slovak Academy of Sciences, Kosice, Slovak Republic; 44 Physics Department, Brookhaven National Laboratory, Upton, NY United States of America; 45 Departamento de Física, Universidad de Buenos Aires, Buenos Aires, Argentina; 46 Cavendish Laboratory, University of Cambridge, Cambridge, UK; 47 Department of Physics, University of Cape Town, Cape Town, South Africa; 48 Department of Mechanical Engineering Science, University of Johannesburg, Johannesburg, South Africa; 49 School of Physics, University of the Witwatersrand, Johannesburg, South Africa; 50 Department of Physics, Carleton University, Ottawa, ON Canada; 51 Faculté des Sciences Ain Chock, Réseau Universitaire de Physique des Hautes Energies, Université Hassan II, Casablanca, Morocco; 52 Centre National de l’Energie des Sciences Techniques Nucleaires (CNESTEN), Rabat, Morocco; 53 Faculté des Sciences Semlalia, Université Cadi Ayyad, LPHEA-Marrakech, Marrakesh, Morocco; 54 Faculté des Sciences, Université Mohamed Premier and LPTPM, Oujda, Morocco; 55 Faculté des sciences, Université Mohammed V, Rabat, Morocco; 56 CERN, Geneva, Switzerland; 57 Enrico Fermi Institute, University of Chicago, Chicago, IL USA; 58 LPC, Université Clermont Auvergne, CNRS/IN2P3, Clermont-Ferrand, France; 59 Nevis Laboratory, Columbia University, Irvington, NY United States of America; 60 Niels Bohr Institute, University of Copenhagen, Copenhagen, Denmark; 61 Dipartimento di Fisica, Università della Calabria, Rende, Italy; 62 INFN Gruppo Collegato di Cosenza, Laboratori Nazionali di Frascati, Frascati, Italy; 63 Physics Department, Southern Methodist University, Dallas, TX United States of America; 64 Physics Department, University of Texas at Dallas, Richardson, TX United States of America; 65 Department of Physics, Stockholm University, Stockholm, Sweden; 66 Oskar Klein Centre, Stockholm, Sweden; 67 Deutsches Elektronen-Synchrotron DESY, Hamburg and Zeuthen, Zeuthen, Germany; 68 Lehrstuhl für Experimentelle Physik IV, Technische Universität Dortmund, Dortmund, Germany; 69 Institut für Kern- und Teilchenphysik, Technische Universität Dresden, Dresden, Germany; 70 Department of Physics, Duke University, Durham, NC United States of America; 71 SUPA - School of Physics and Astronomy, University of Edinburgh, Edinburgh, United Kingdom; 72 INFN e Laboratori Nazionali di Frascati, Frascati, Italy; 73 Physikalisches Institut, Albert-Ludwigs-Universität Freiburg, Freiburg, Germany; 74 II Physikalisches Institut, Georg-August-Universität Göttingen, Göttingen, Germany; 75 Département de Physique Nucléaire et Corpusculaire, Université de Genève, Geneva, Switzerland; 76 Dipartimento di Fisica, Università di Genova, Genova, Italy; 77 INFN Sezione di Genova, Genova, Italy; 78 II. Physikalisches Institut, Justus-Liebig-Universität Giessen, Giessen, Germany; 79 SUPA - School of Physics and Astronomy, University of Glasgow, Glasgow, United Kingdom; 80 LPSC, Université Grenoble Alpes, CNRS/IN2P3, Grenoble INP, Grenoble, France; 81 Laboratory for Particle Physics and Cosmology, Harvard University, Cambridge, MA United States of America; 82 Department of Modern Physics and State Key Laboratory of Particle Detection and Electronics, University of Science and Technology of China, Hefei, China; 83 Institute of Frontier and Interdisciplinary Science and Key Laboratory of Particle Physics and Particle Irradiation (MOE), Shandong University, Qingdao, China; 84 School of Physics and Astronomy, Shanghai Jiao Tong University, KLPPAC-MoE, SKLPPC, Shanghai, China; 85 Tsung-Dao Lee Institute, Shanghai, China; 86 Kirchhoff-Institut für Physik, Ruprecht-Karls-Universität Heidelberg, Heidelberg, Germany; 87 Physikalisches Institut, Ruprecht-Karls-Universität Heidelberg, Heidelberg, Germany; 88 Faculty of Applied Information Science, Hiroshima Institute of Technology, Hiroshima, Japan; 89 Department of Physics, Chinese University of Hong Kong, Shatin, N.T. Hong Kong; 90 Department of Physics, University of Hong Kong, Hong Kong, China; 91 Department of Physics and Institute for Advanced Study, Hong Kong University of Science and Technology, Clear Water Bay, Kowloon, Hong Kong, China; 92 Department of Physics, National Tsing Hua University, Hsinchu, Taiwan; 93 Department of Physics, Indiana University, Bloomington, IN USA; 94 INFN Gruppo Collegato di Udine, Sezione di Trieste, Udine, Italy; 95 ICTP, Trieste, Italy; 96 Dipartimento di Chimica, Fisica e Ambiente, Università di Udine, Udine, Italy; 97 INFN Sezione di Lecce, Zona Monte, Italy; 98 Dipartimento di Matematica e Fisica, Università del Salento, Lecce, Italy; 99 INFN Sezione di Milano, Milan, Italy; 100 Dipartimento di Fisica, Università di Milano, Milan, Italy; 101 INFN Sezione di Napoli, Napoli, Italy; 102 Dipartimento di Fisica, Università di Napoli, Napoli, Italy; 103 INFN Sezione di Pavia, Pavia, Italy; 104 Dipartimento di Fisica, Università di Pavia, Pavia, Italy; 105 INFN Sezione di Pisa, Pisa, Italy; 106 Dipartimento di Fisica E. Fermi, Università di Pisa, Pisa, Italy; 107 INFN Sezione di Roma, Rome, Italy; 108 Dipartimento di Fisica, Sapienza Università di Roma, Rome, Italy; 109 INFN Sezione di Roma Tor Vergata, Rome, Italy; 110 Dipartimento di Fisica, Università di Roma Tor Vergata, Rome, Italy; 111 INFN Sezione di Roma Tre, Rome, Italy; 112 Dipartimento di Matematica e Fisica, Università Roma Tre, Rome, Italy; 113 INFN-TIFPA, Povo, Italy; 114 Università degli Studi di Trento, Trento, Italy; 115 Institut für Astro- und Teilchenphysik, Leopold-Franzens-Universität, Innsbruck, Austria; 116 University of Iowa, Iowa City, IA USA; 117 Department of Physics and Astronomy, Iowa State University, Ames, IA USA; 118 Joint Institute for Nuclear Research, Dubna, Russia; 119 Departamento de Engenharia Elétrica, Universidade Federal de Juiz de Fora (UFJF), Juiz de Fora, Brazil; 120 Universidade Federal do Rio De Janeiro COPPE/EE/IF, Rio de Janeiro, Brazil; 121 Universidade Federal de São João del Rei (UFSJ), São João del Rei, Brazil; 122 Instituto de Física, Universidade de São Paulo, São Paulo, Brazil; 123 KEK, High Energy Accelerator Research Organization, Tsukuba, Japan; 124 Graduate School of Science, Kobe University, Kobe, Japan; 125 Faculty of Physics and Applied Computer Science, AGH University of Science and Technology, Krakow, Poland; 126 Marian Smoluchowski Institute of Physics, Jagiellonian University, Krakow, Poland; 127 Institute of Nuclear Physics Polish Academy of Sciences, Krakow, Poland; 128 Faculty of Science, Kyoto University, Kyoto, Japan; 129 Kyoto University of Education, Kyoto, Japan; 130 Research Center for Advanced Particle Physics and Department of Physics, Kyushu University, Fukuoka, Japan; 131 Instituto de Física La Plata, Universidad Nacional de La Plata and CONICET, La Plata, Argentina; 132 Physics Department, Lancaster University, Lancaster, UK; 133 Oliver Lodge Laboratory, University of Liverpool, Liverpool, UK; 134 Department of Experimental Particle Physics, Jožef Stefan Institute and Department of Physics, University of Ljubljana, Ljubljana, Slovenia; 135 School of Physics and Astronomy, Queen Mary University of London, London, UK; 136 Department of Physics, Royal Holloway University of London, Egham, UK; 137 Department of Physics and Astronomy, University College London, London, UK; 138 Louisiana Tech University, Ruston, LA USA; 139 Fysiska institutionen, Lunds universitet, Lund, Sweden; 140 Centre de Calcul de l’Institut National de Physique Nucléaire et de Physique des Particules (IN2P3), Villeurbanne, France; 141 Departamento de Física Teorica C-15 and CIAFF, Universidad Autónoma de Madrid, Madrid, Spain; 142 Institut für Physik, Universität Mainz, Mainz, Germany; 143 School of Physics and Astronomy, University of Manchester, Manchester, UK; 144 CPPM, Aix-Marseille Université, CNRS/IN2P3, Marseille, France; 145 Department of Physics, University of Massachusetts, Amherst, MA USA; 146 Department of Physics, McGill University, Montreal, QC Canada; 147 School of Physics, University of Melbourne, Melbourne, VIC Australia; 148 Department of Physics, University of Michigan, Ann Arbor, MI USA; 149 Department of Physics and Astronomy, Michigan State University, East Lansing, MI USA; 150 B.I. Stepanov Institute of Physics, National Academy of Sciences of Belarus, Minsk, Belarus; 151 Research Institute for Nuclear Problems of Byelorussian State University, Minsk, Belarus; 152 Group of Particle Physics, University of Montreal, Montreal, QC Canada; 153 P.N. Lebedev Physical Institute of the Russian Academy of Sciences, Moscow, Russia; 154 Institute for Theoretical and Experimental Physics (ITEP), Moscow, Russia; 155 National Research Nuclear University MEPhI, Moscow, Russia; 156 D.V. Skobeltsyn Institute of Nuclear Physics, M.V. Lomonosov Moscow State University, Moscow, Russia; 157 Fakultät für Physik, Ludwig-Maximilians-Universität München, Munich, Germany; 158 Max-Planck-Institut für Physik (Werner-Heisenberg-Institut), Munich, Germany; 159 Nagasaki Institute of Applied Science, Nagasaki, Japan; 160 Graduate School of Science and Kobayashi-Maskawa Institute, Nagoya University, Nagoya, Japan; 161 Department of Physics and Astronomy, University of New Mexico, Albuquerque, NM USA; 162 Institute for Mathematics, Astrophysics and Particle Physics, Radboud University Nijmegen/Nikhef, Nijmegen, Netherlands; 163 Nikhef National Institute for Subatomic Physics, University of Amsterdam, Amsterdam, Netherlands; 164 Department of Physics, Northern Illinois University, DeKalb, IL USA; 165 Budker Institute of Nuclear Physics, SB RAS, Novosibirsk, Russia; 166 Novosibirsk State University, Novosibirsk, Russia; 167 Department of Physics, New York University, New York, NY USA; 168 Ohio State University, Columbus, OH USA; 169 Faculty of Science, Okayama University, Okayama, Japan; 170 Homer L. Dodge Department of Physics and Astronomy, University of Oklahoma, Norman, OK USA; 171 Department of Physics, Oklahoma State University, Stillwater, OK USA; 172 Palacký University, RCPTM, Joint Laboratory of Optics, Olomouc, Czech Republic; 173 Center for High Energy Physics, University of Oregon, Eugene, OR USA; 174 LAL, Université Paris-Sud, CNRS/IN2P3, Université Paris-Saclay, Orsay, France; 175 Graduate School of Science, Osaka University, Osaka, Japan; 176 Department of Physics, University of Oslo, Oslo, Norway; 177 Department of Physics, Oxford University, Oxford, UK; 178 LPNHE, Sorbonne Université, Paris Diderot Sorbonne Paris Cité, CNRS/IN2P3 Paris, France; 179 Department of Physics, University of Pennsylvania, Philadelphia, PA USA; 180 Konstantinov Nuclear Physics Institute of National Research Centre “Kurchatov Institute”, PNPI, St. Petersburg, Russia; 181 Department of Physics and Astronomy, University of Pittsburgh, Pittsburgh, PA USA; 182 Laboratório de Instrumentação e Física Experimental de Partículas-LIP, Lisbon, Portugal; 183 Departamento de Física, Faculdade de Ciências, Universidade de Lisboa, Lisbon, Portugal; 184 Departamento de Física, Universidade de Coimbra, Coimbra, Portugal; 185 Centro de Física Nuclear da Universidade de Lisboa, Lisbon, Portugal; 186 Departamento de Física, Universidade do Minho, Braga, Portugal; 187 Departamento de Física Teorica y del Cosmos, Universidad de Granada, Granada, Spain; 188 Dep Física and CEFITEC of Faculdade de Ciências e Tecnologia, Universidade Nova de Lisboa, Caparica, Portugal; 189 Institute of Physics, Academy of Sciences of the Czech Republic, Prague, Czech Republic; 190 Czech Technical University in Prague, Prague, Czech Republic; 191 Faculty of Mathematics and Physics, Charles University, Prague, Czech Republic; 192 State Research Center Institute for High Energy Physics, NRC KI, Protvino, Russia; 193 Particle Physics Department, Rutherford Appleton Laboratory, Didcot, UK; 194 IRFU, CEA , Université Paris-Saclay, Gif-sur-Yvette, France; 195 Santa Cruz Institute for Particle Physics, University of California Santa Cruz, Santa Cruz, CA USA; 196 Departamento de Física, Pontificia Universidad Católica de Chile, Santiago, Chile; 197 Departamento de Física, Universidad Técnica Federico Santa María, Valparaíso, Chile; 198 Department of Physics, University of Washington, Seattle, WA USA; 199 Department of Physics and Astronomy, University of Sheffield, Sheffield, UK; 200 Department of Physics, Shinshu University, Nagano, Japan; 201 Department Physik, Universität Siegen, Siegen, Germany; 202 Department of Physics, Simon Fraser University, Burnaby, BC Canada; 203 SLAC National Accelerator Laboratory, Stanford, CA USA; 204 Physics Department, Royal Institute of Technology, Stockholm, Sweden; 205 Departments of Physics and Astronomy, Stony Brook University, Stony Brook, NY USA; 206 Department of Physics and Astronomy, University of Sussex, Brighton, UK; 207 School of Physics, University of Sydney, Sydney, Australia; 208 Institute of Physics, Academia Sinica, Taipei, Taiwan; 209 Academia Sinica Grid Computing, Institute of Physics, Academia Sinica, Taipei, Taiwan; 210 E. Andronikashvili Institute of Physics, Iv. Javakhishvili Tbilisi State University, Tbilisi, Georgia; 211 High Energy Physics Institute, Tbilisi State University, Tbilisi, Georgia; 212 Department of Physics, Technion: Israel Institute of Technology, Haifa, Israel; 213 Raymond and Beverly Sackler School of Physics and Astronomy, Tel Aviv University, Tel Aviv, Israel; 214 Department of Physics, Aristotle University of Thessaloniki, Thessaloniki, Greece; 215 International Center for Elementary Particle Physics and Department of Physics, University of Tokyo, Tokyo, Japan; 216 Graduate School of Science and Technology, Tokyo Metropolitan University, Tokyo, Japan; 217 Department of Physics, Tokyo Institute of Technology, Tokyo, Japan; 218 Tomsk State University, Tomsk, Russia; 219 Department of Physics, University of Toronto, Toronto, ON Canada; 220 TRIUMF, Vancouver, BC Canada; 221 Department of Physics and Astronomy, York University, Toronto, ON Canada; 222 Division of Physics and Tomonaga Center for the History of the Universe, Faculty of Pure and Applied Sciences, University of Tsukuba, Tsukuba, Japan; 223 Department of Physics and Astronomy, Tufts University, Medford, MA USA; 224 Department of Physics and Astronomy, University of California Irvine, Irvine, CA USA; 225 Department of Physics and Astronomy, University of Uppsala, Uppsala, Sweden; 226 Department of Physics, University of Illinois, Urbana, IL USA; 227 Instituto de Física Corpuscular (IFIC), Centro Mixto Universidad de Valencia - CSIC, Valencia, Spain; 228 Department of Physics, University of British Columbia, Vancouver, BC Canada; 229 Department of Physics and Astronomy, University of Victoria, Victoria, BC Canada; 230 Fakultät für Physik und Astronomie, Julius-Maximilians-Universität Würzburg, Würzburg, Germany; 231 Department of Physics, University of Warwick, Coventry, UK; 232 Waseda University, Tokyo, Japan; 233 Department of Particle Physics, Weizmann Institute of Science, Rehovot, Israel; 234 Department of Physics, University of Wisconsin, Madison, WI USA; 235 Fakultät für Mathematik und Naturwissenschaften, Fachgruppe Physik, Bergische Universität Wuppertal, Wuppertal, Germany; 236 Department of Physics, Yale University, New Haven, CT USA; 237 Yerevan Physics Institute, Yerevan, Armenia; 238 CERN, 1211 Geneva 23, Switzerland

## Abstract

Previous studies have shown that weighted angular moments derived from jet constituents encode the colour connections between partons that seed the jets. This paper presents measurements of two such distributions, the jet-pull angle and jet-pull magnitude, both of which are derived from the jet-pull angular moment. The measurement is performed in $t\bar{t}$ events with one leptonically decaying *W* boson and one hadronically decaying *W* boson, using $36.1\,\text {fb}^{-1}$ of *pp* collision data recorded by the ATLAS detector at $\sqrt{s} = 13 \, \text {TeV}$ delivered by the Large Hadron Collider. The observables are measured for two dijet systems, corresponding to the colour-connected daughters of the *W* boson and the two *b*-jets from the top-quark decays, which are not expected to be colour connected. To allow the comparison of the measured distributions to colour model predictions, the measured distributions are unfolded to particle level, after correcting for experimental effects introduced by the detector. While good agreement can be found for some combinations of predictions and observables, none of the predictions describes the data well across all observables.

## Introduction

**Fig. 1:**
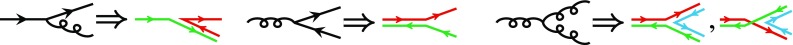
QCD colour propagation rules for elementary quark–gluon vertices. Black lines denote Feynman-diagram style vertices, coloured lines show QCD colour connection lines

In high-energy hadron collisions, such as those produced at the Large Hadron Collider (LHC) [1] at CERN, quarks and gluons are produced abundantly. However, due to the confining nature of quantum chromodynamics (QCD), the direct measurement of the interactions that occur between these particles is impossible and only colour-neutral hadrons can be measured. To a good approximation, the radiation pattern in QCD can be described through a colour–connection picture, which consists of colour strings connecting quarks and gluons of one colour to quarks and gluons of the corresponding anti–colour. Figure 1 illustrates the colour connections for the relevant elementary QCD vertices.

In the decay chain of a hard-scatter event, the colour charge “flows” from the initial state towards stable particles whilst following the rules illustrated in Fig. 1. As colour charge is conserved, connections exist between initial particles and the stable colour-neutral hadrons.

In practice, high-energy quarks and gluons are measured as jets, which are bunches of collimated hadrons that form in the evolution of the coloured initial particles. The colour connections between high-energy particles affect the structure of the emitted radiation and therefore also the structure of the resulting jets. For example, soft gluon radiation is suppressed in some regions of phase space compared to others. Specifically, due to colour coherence effects, QCD predicts an increase of radiation where a colour connection is present compared to a region of phase space where no such connection exists, see Ref. [2]. Smaller effects on the event topology and measured quantities are expected from colour reconnection in the hadronisation process.

Providing evidence for the existence of the connections between particles – the *colour flow* – is important for the validation of phenomenological descriptions. Using the energy-weighted distributions of particles within and between jets has been a long-standing tool for investigating colour flow, with early measurements at PETRA [3] and LEP [4, 5]. Later, a precursor of the jet pull was studied using the abundant jet production at the Tevatron [6]. Recently, the colour flow was measured by ATLAS in $t\bar{t}$ events at the LHC at a centre-of-mass energy of $\sqrt{s}=8\,\hbox {TeV}$ [7] using the jet-pull angle.

**Fig. 2:**
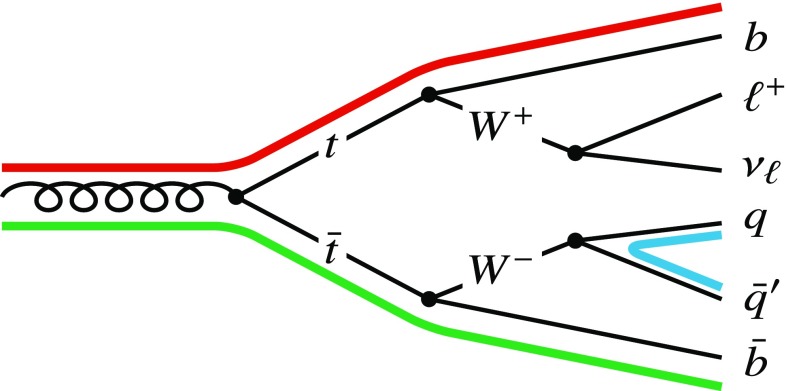
Illustration of a semileptonic $t\bar{t}$ event with typical colour connections (thick coloured lines)

Figure 2 illustrates the production of a $t\bar{t}$ pair and its subsequent decay into a single-lepton final state as produced at the LHC with colour connections superimposed. In the hard-scatter event, four colour-charged final states can be identified: the two *b*-quarks produced directly by the decay of the top-quarks and the two quarks produced by the hadronically decaying *W* boson. As the *W* boson does not carry colour charge, its daughters must share a colour connection. The two *b*-quarks from the top-quark decays carry the colour charge of their respective top-quark parent, and are thus not expected to share a colour connection.

Despite the long-standing history of measurements of the potential effects of colour connections, they remain a poorly constrained effect of QCD and require further experimental input. Furthermore, it may be possible to use the extracted colour information to distinguish between event topologies with a different colour structure. In the case of jets, such colour information would complement the kinematic properties, and might enable the identification of otherwise irreducible backgrounds, or facilitate the correct assignment of jets to a particular physical process. For example, a colour-flow observable could be used to resolve the ambiguity in assigning *b*-jets to the Higgs boson decay in $t\bar{t}H(\rightarrow b\bar{b})$ events.

An observable predicted to encode colour information about a jet is the jet-pull vector $\vec {\mathcal {P}}$ [8], a $p_{{\text {T}}}$-weighted radial moment of the jet. For a given jet *j* with transverse momentum $p_{{\text {T}}} ^j$, the observable is defined as

1 $$\begin{aligned} \vec {\mathcal {P}}\left( j \right) = \sum _{i \in j} \frac{\left| \vec {\Delta r}_{i} \right| \cdot p_{{\text {T}}} ^i}{p_{{\text {T}}} ^j} \vec {\Delta r}_i, \end{aligned}$$

where the summation runs over the constituents of *j* that have transverse momentum $p_{{\text {T}}} ^i$ and are located at $\vec {\Delta r}_i = \left( \Delta y_i, \Delta \phi _i \right)$, which is the offset of the constituent from the jet axis $(y_j, \phi _j)$ in rapidity–azimuth (*y*–$\phi$) space.^1^ Examples of constituents that could be used in Eq. (1) include calorimeter energy clusters, inner-detector tracks, and simulated stable particles.

**Fig. 3:**
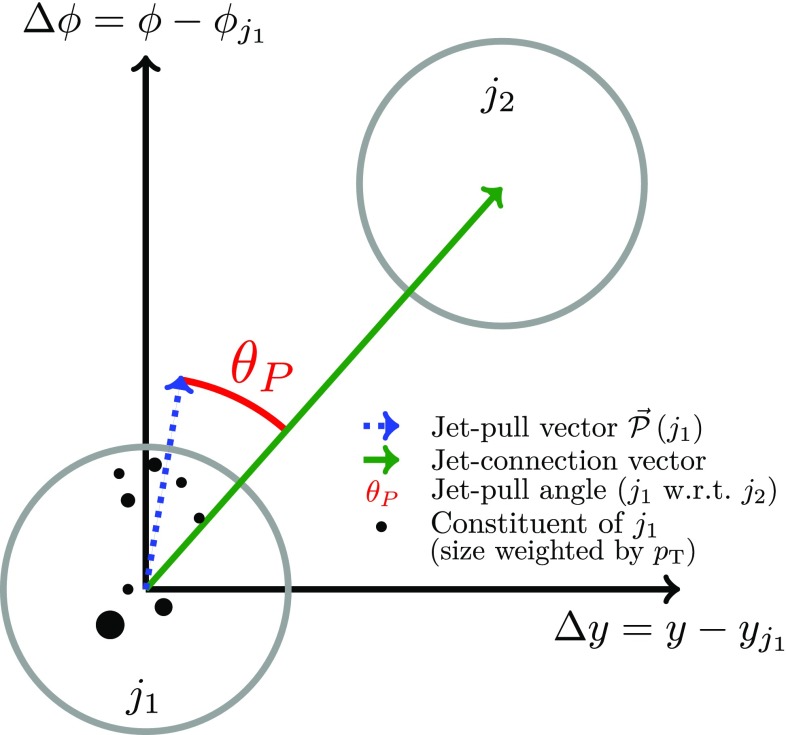
Illustration of jet-pull observables for a dijet system. For a jet $j_1$ the jet-pull vector is calculated using an appropriate set of constituents (tracks, calorimeter energy clusters, simulated particles, ...). The variable of particular sensitivity to the colour structure of $j_1$ with respect to $j_2$ is the jet-pull angle $\theta _P$ which is the angle between the pull vector for $j_1$ and the vector connecting $j_1$ to another jet $j_2$ in localised *y*–$\phi$ space (the “jet connection vector”)

Given two jets, $j_1$ and $j_2$, the jet-pull vector can be used to construct the jet-pull angle $\theta _{\mathcal {P}}\left( j_1, j_2 \right)$. This is defined as the angle between the jet-pull vector $\vec {\mathcal {P}}\left( j_1 \right)$ and the vector connecting $j_1$ to $j_2$ in rapidity–azimuth space, $\left( y_{j_2} - y_{j_1}, \phi _{j_2} - \phi _{j_1} \right)$, which is called “jet connection vector”. Figure 3 illustrates the jet-pull vector and angle for an idealised dijet system. As the jet-pull angle is symmetric around zero and takes values ranging from $-\pi$ to $\pi$, it is convenient to consider the normalised absolute pull angle $\left| \theta _{\mathcal {P}} \right| / \pi$ as the observable. The measurement presented here is performed using this normalisation.

The jet-pull angle is particularly suited for studying the colour structure of an object decaying to a dijet system, as the inputs into the calculation are well-defined theoretically and the observable is expected to be sensitive to the presence or absence of a colour connection. For two colour-connected jets, $j_1$ and $j_2$, it is expected that $\vec {\mathcal {P}}\left( j_1 \right)$ and $\vec {\mathcal {P}}\left( j_2 \right)$ are aligned with the jet connection vector, i.e. $\theta _{\mathcal {P}} \sim 0$. For two jets without any particular colour connection, the jet-pull vector and the connection vector are not expected to be aligned and thus $\theta _{\mathcal {P}}$ is expected to be distributed uniformly.

In this paper, the normalised jet-pull angle is measured for two different systems of dijets in $t\bar{t}$ events using $36.1\,\text {fb}^{-1}$ of *pp* collision data recorded by the ATLAS detector at $\sqrt{s} = 13 \, \text {TeV}$. The first targets the jets originating from the hadronic decay of a *W* boson and thus from a colour singlet, while the second targets the two *b*-jets from the top decays, which are not expected to be colour connected. The magnitude of the jet-pull vector is also measured. The results are presented as normalised distributions corrected for detector effects.

In Sect. 2, the ATLAS detector is introduced. Section 3 discusses the data and simulation samples used by this analysis. The reconstruction procedures and event selection are presented in Sect. 4. In Sect. 5 the analysis observables are introduced and discussed in detail. Section 6 introduces the phase space of the particle-level measurement and the unfolding procedure used to correct the observed data for detector effects. In Sect. 7 the relevant uncertainties and the methodology used to assess them are discussed. Finally, Sect. 8 presents the results, followed by a conclusion in Sect. 9.

## The ATLAS detector

The ATLAS detector [9] is a multi-purpose detector with a near $4\pi$ coverage in solid angle. It uses a system of tracking detectors, which enclose the interaction point, to provide highly resolved spatial measurements of charged particles in the range $\left| \eta \right| < 2.5$. These tracking detectors, collectively called the inner detector, are immersed in a 2 T magnetic field enabling reconstruction of the track momentum. During the Long Shutdown 1, a new innermost layer of the pixel detector was inserted into the detector, the insertable B-layer (IBL) [10, 11]. Two calorimeter subsystems enclose the inner detector allowing complementary calorimetric measurements of both the charged and neutral particles. Behind the calorimeters a system of muon chambers provides muon identification, triggering, and (additional) tracking. The muon system is immersed in a magnetic field provided by three toroid magnets. A more complete description of the ATLAS detector can be found elsewhere [9].

Data are selected for read-out and further processing by a two-stage trigger [12] that uses coarse detector information in a hardware-based first stage followed by a software-based second trigger stage, which has access to the full detector granularity. This reduces the raw rate of 40 MHz from the LHC *pp* collisions to about 75 kHz after the first stage and 1 kHz after the second stage.

## Data sample and simulation

The data used by this analysis were collected in 2015 and 2016 during *pp* runs provided by the LHC at a centre-of-mass energy of $\sqrt{s}=13\,\hbox {TeV}$. Stable beams and fully operational subdetectors are required. After data quality requirements, the data correspond to an integrated luminosity of $\mathcal {L}_{{\text {Int}}} = 36.1\,\hbox {fb}^{-1}$.

Monte Carlo (MC) samples are used to evaluate the contribution of background processes to the selected event sample, evaluate how the detector response affects the analysis observables and for comparisons with the measured data. A variety of configurations are investigated for different purposes. Table 1 summarises the samples used by the analysis.

The $t\bar{t}$ sample in the first row of the table (the *“nominal”* sample) is used to evaluate how well the data agrees with MC simulation, predict the number of signal events, and obtain the nominal detector response description. This sample was generated using the Powheg-Box  v2 [13–15] event generator with the NNPDF 3.0 parton distribution functions (PDF) [16]. The top-quark mass, $m_t$, was set to $172.5\,\hbox {GeV}$ and the value of the $h_{\mathrm {damp}}$ parameter, which controls the $p_{{\text {T}}}$ of the first emission beyond the Born configuration in Powheg, was set to $1.5~m_t$. The main effect of $h_{\mathrm {damp}}$ is to regulate the high-$p_{{\text {T}}}$ emission against which the $t\bar{t}$ system recoils. Pythia 8  [17] with the NNPDF 2.3 [18] PDF set and the A14 [19] tune^2^ was used to simulate the parton shower, hadronisation and underlying event.

**Table 1:** Monte Carlo samples used for this analysis. The first part of the table shows samples generated for the signal process, the second those for processes considered to be a background. Samples / tunes marked with $\dagger$ refer to alternative signal MC samples used to evaluate signal modelling uncertainties, those marked with $\star$ are used for comparison to the measurement result. The default *tW*-channel single-top MC sample is generated using the “diagram removal” scheme [31]. The following abbreviations are used: *ME* matrix element, *PS* parton shower, *LO* leading-order calculation in QCD, *NLO* next-to-leading-order calculation in QCD, *PDF* parton distribution function

**Process**	**Generator**	**Type**	**Version**	**PDF**	**Tune** $^{2}$
$t\bar{t}$	Powheg-Box v2 [13–15]	NLO ME	r3026	NNPDF 3.0 [16]	–
	+Pythia 8 [17]	+LO PS	v8.186	NNPDF 2.3 [18]	A14 / A14.v1 $^{\dagger }$ / A14.v3c $^{\dagger }$ [19]
$t\bar{t}^{\dagger }$	Powheg-Box v2	NLO ME	r3026	NNPDF 3.0	–
	+Herwig 7 [20]	+LO PS	v7.0.1.a	MMHT 2014 [21]	H7UE
$t\bar{t}^{\dagger }$	MadGraph5_aMC@NLO [22]	NLO ME	v2.3.3.p1	NNPDF 3.0	–
	+Pythia 8	+LO PS	v8.112	NNPDF 2.3	A14
$t\bar{t}^{\star }$	Powheg-Box v2	NLO ME	r2819	CT10 [23]	–
	+Pythia 6 [24]	+LO PS	v6.428	CTEQ6L1 [25]	Perugia 2012 [26]
$t\bar{t}^{\star }$	Sherpa [27–29]	LO/NLO multileg ME+PS	v2.2.1	NNPDF 3.0 NNLO	–
Single top	Powheg-Box v1	NLO ME	r2819	CT10 (5FS)	–
(*t*-, *s*-, *tW*-channel)	+Pythia 6	+LO PS	v6.425	CTEQ6L1	Perugia 2012
$WW,\,WZ,\,ZZ$	Sherpa	LO/NLO multileg ME+PS	v2.1.1	CT10	Default
$W/Z+\text {jets}$	Sherpa	LO/NLO multileg ME+PS	v2.2.1	NNPDF 3.0	Default
$t\bar{t}W/Z$	MadGraph5_aMC@NLO	NLO ME	v2.3.3	NNPDF 3.0	–
	+Pythia 8 [30]	+LO PS	v8.210	NNPDF 2.3	A14
$t\bar{t}H$	MadGraph5_aMC@NLO	NLO ME	v2.2.3.p4	NNPDF 3.0	–
	+Pythia 8	+LO PS	v8.210	NNPDF 2.3	A14

To evaluate the impact of systematic uncertainties coming from signal modelling on the measurements, a variety of alternative signal MC samples are used. These samples or tunes are marked with a $\dagger$ in Table 1. To assess the impact of increased or reduced radiation, samples were generated using the A14.v3c up and down tune variations. Additionally, in the A14.v3c up (down) variation sample the renormalisation and factorisation scales were scaled by a factor of 0.5 (2) relative to the nominal sample and the value of $h_{{\mathrm {damp}}}$ was set to $3 m_t$ ($1.5 m_t$) [32]. Similarly, to assess the impact of colour reconnection, two samples generated with the A14.v1 tune variations are used. These modify simulation parameters which configure the strong coupling of multi-parton interactions and the strength of the colour-reconnection mechanism [19]. Two alternative MC programs are used in order to estimate the impact of the choice of hard-scatter generator and hadronisation algorithm: for each of these samples one of the two components is replaced by an alternative choice. The alternative choices are MadGraph5_aMC@NLO  (MG5_aMC) [22] for the hard-scatter generator and Herwig 7  [20] for the hadronisation algorithm.

Two additional simulation set-ups are used to obtain $t\bar{t}$ predictions, both of which are marked with a $\star$ in Table 1: one sample uses Powheg-Box  v2, with $h_{\mathrm {damp}}$ set to the top-quark mass, interfaced to Pythia 6 for the hadronisation and parton shower, using the Perugia 2012 tune [26]. The second set-up uses the Sherpa  [27–29] MC program with a parton shower tune developed by the Sherpa authors.

Signal MC simulation is normalised to a theoretical cross-section of $832^{+46}_{-51} \, \hbox {pb}$, where the uncertainties reflect the effect of scale, PDF, and $\alpha _s$ variations as well as the top-quark mass uncertainty. This is calculated with the Top++ 2.0 program [33] to next-to-next-to-leading order in perturbative QCD, including resummation of next-to-next-to-leading-logarithm soft-gluon terms, assuming a top-quark mass of $172.5\,\hbox {GeV}$ [34–39]. Normalised signal MC simulation is only used to compare the observed data to the prediction.

Contributions from processes considered to be a background to the analysis are in most cases modelled using simulation samples. These samples are shown in the second part of Table 1. All background MC samples are normalised to their theoretical cross-sections evaluated to at least next-to-leading order (NLO) precision in QCD [40–47, 47, 48].

Multiple overlaid *pp* collisions, which are causing so called pile-up, were simulated with the soft QCD processes of Pythia 8.186  [17] using the A2 [49] tune and the MSTW2008LO PDF set [50]. A reweighting procedure was applied on an event-by-event basis to the simulation samples to reflect the distribution of the average number of *pp* interactions per event observed in data.

Events generated by the MC programs are further processed using the ATLAS detector and trigger simulation [51] which uses Geant4 [52] to simulate the interactions between particles and the detector material. The samples used to evaluate the detector response and estimate the background contributions were processed using the full ATLAS simulation [51]. Alternative signal MC samples, which are used to evaluate signal modelling uncertainties, were processed using Atlfast II [53]. This detector simulation differs from the full ATLAS detector simulation by using a faster method to model energy depositions in the calorimeter, while leaving the simulation of the remainder of the detector unchanged. The results of this analysis are found to be consistent when using either full ATLAS simulation or Atlfast II simulation.

In order to evaluate the sensitivity of the analysis observables to colour flow and to be able to assess the colour-model dependence of the analysis methods, a dedicated MC sample with a simulated exotic colour-flow model is used; this is labelled as *“(colour) flipped”*. In this sample, the colour-singlet *W* boson in ordinary signal events is replaced *ad hoc* by a colour octet. To create this sample, hard-scatter signal events were generated using Powheg-Box  v2 with the same settings as the nominal $t\bar{t}$ sample and stored in the LHE format [54]. The colour strings were then flipped in such a way that, among the decay products obtained from the hadronic decay of the *W* boson, one of them is connected to the incoming top quark while the other one is connected to the outgoing *b*-quark. Pythia 8 was then used to perform the showering and hadronisation in the modified hard-scatter event using the same procedure as in the nominal $t\bar{t}$ sample.

## Event reconstruction and selection

In order to have a dataset that is enriched in events with a hadronically decaying *W* boson, and in which the resulting jets can be identified with reasonable accuracy, this analysis targets the $t\bar{t} \rightarrow b\bar{b}W(\rightarrow \ell \nu )W(\rightarrow q\bar{q}^{\prime })$ final state, where $\ell$ refers to electrons and muons.^3^ Such a sample provides access to both a pair of colour-connected ($q\bar{q}^{\prime }$) and non-connected ($b\bar{b}$) jets.

In the following, the definitions used for the object reconstruction, as well as the event selection used to obtain a signal-enriched sample in data, are discussed.

### Detector-level objects

Primary vertices are constructed from all reconstructed tracks compatible with the interaction region given by LHC beam-spot characteristics [55]. The hard-scatter primary vertex is then selected as the vertex with the largest $\sum p_{{\text {T}}} ^2$, where tracks entering the summation must satisfy $p_{{\text {T}}} > 0.4\,\hbox {GeV}$.

Candidate electrons are reconstructed by matching tracks from the inner detector to energy deposits in the electromagnetic calorimeter. Electron identification (ID) relies on a likelihood classifier constructed from various detector inputs such as calorimeter shower shape or track quality [56–58]. The electron candidates must satisfy a “tight” ID criterion as defined in Ref. [58]. They must further satisfy $E_{\mathrm {T}} > 25\,\hbox {GeV}$ and $\left| \eta \right| < 2.47$, with the region $1.37 \le \left| \eta \right| \le 1.52$ being excluded. This is the transition region between the barrel and endcap of the electromagnetic calorimeter, and as a result the energy resolution is significantly degraded within this region. Isolation requirements using calorimeter and tracking requirements are applied to reduce background from non-prompt and fake electrons [59]. The resulting isolation efficiency increases linearly with the electron $p_{{\text {T}}}$, starting at approximately 90% and reaching a plateau of 99% at approximately $p_{{\text {T}}} = 60\,\hbox {GeV}$. Electrons are also required to have $|d_0^{{\text {sig}}}| < 5$ and $|z_0 \sin {\theta }| < 0.5\,\hbox {mm}$, where $|d_0^{{\text {sig}}}| = |d_0|/\sigma _{d_0}$ is the significance of the transverse impact parameter relative to the beamline, and $z_0$ is the distance along the *z*-axis from the primary vertex to the point where the track is closest to the beamline.

Muon candidates are reconstructed by matching tracks in the muon spectrometer to inner-detector tracks. Muons must satisfy the “medium” ID criteria and the “gradient” isolation criteria as defined in Ref. [60]. The muon $p_{{\text {T}}}$ is determined from a fit of all hits associated with the muon track, also taking into account the energy loss in the calorimeters. Furthermore, muons must satisfy $p_{{\text {T}}} > 25\,\hbox {GeV}$ and $\left| \eta \right| < 2.5$. Finally, muon tracks must have $|d_0^{{\text {sig}}}| < 3$ and $|z_0 \sin \theta | < 0.5\,\hbox {mm}$.

Jets are reconstructed using the anti-$k_t$ algorithm [61] with radius parameter $R=0.4$ as implemented by the FastJet [62] package. The inputs to the jet algorithm consist of three-dimensional, massless, positive-energy topological clusters [63, 64] constructed from energy deposited in the calorimeters. The jet four-momentum is calibrated using an $\eta$- and energy-dependent scheme with *in situ* corrections based on data [65, 66]. The calibrated four-momentum is required to satisfy $p_{{\text {T}}} > 25\,\hbox {GeV}$ and $\left| \eta \right| < 2.5$. To reduce the number of jets originating from pile-up, an additional selection criterion based on a jet-vertex tagging technique [67] is applied to jets with $p_{{\text {T}}} < 60\,\hbox {GeV}$ and $\left| \eta \right| < 2.4$. A multivariate discriminant is used to identify jets containing *b*-hadrons, using track impact parameters, track invariant mass, track multiplicity and secondary-vertex information. The *b*-tagging algorithm [68, 69] is used at a working point that is constructed to operate at an overall *b*-tagging efficiency of 70% in simulated $t\bar{t}$ events for jets with $p_{{\text {T}}} > 20\,\hbox {GeV}$. The corresponding *c*-jet and light-jet rejection factors are 12 and 381 respectively, resulting in a purity of 97%.

Detector information may produce objects that satisfy both the jet and lepton criteria. In order to match the detector information to a unique physics object, an overlap removal procedure is applied: double-counting of electron energy deposits as jets is prevented by discarding the closest jet lying a distance $\Delta R < 0.2$ from a reconstructed electron. Subsequently, if an electron lies $\Delta R < 0.4$ from a jet, the electron is discarded in order to reduce the impact of non-prompt leptons. Furthermore, if a jet has fewer than three associated tracks and lies $\Delta R < 0.4$ from a muon, the jet is discarded. Conversely, any muon that lies $\Delta R < 0.4$ from a jet with at least three associated tracks is discarded.

The magnitude of the missing transverse momentum $E_{{\text {T}}}^{{\text {miss}}}$ is calculated as the transverse component of the negative vector sum of the calibrated momentum of all objects in the event [70, 71]. This sum includes contributions from soft, non-pile-up tracks not associated with any of the physics objects discussed above.

### Event selection

Firstly, basic event-level quality criteria are applied, such as the presence of a primary vertex and the requirement of stable detector conditions. Then, events are selected by requiring that a single-electron or single-muon trigger has fired. The triggers are designed to select well-identified charged leptons with high $p_{{\text {T}}}$. They require a $p_{{\text {T}}}$ of at least 20 (26) GeV for muons and 24 (26) GeV for electrons for the 2015 (2016) data set and also include requirements on the lepton quality and isolation. These triggers are complemented by triggers with higher $p_{{\text {T}}}$ requirements but loosened isolation and identification requirements to ensure maximum efficiencies at higher lepton $p_{{\text {T}}}$.

The reconstructed lepton must satisfy $p_{{\text {T}}} > 27\,\hbox {GeV}$ and must match the trigger-level object that fired using a geometrical matching. No additional lepton with $p_{{\text {T}}} > 25\,\hbox {GeV}$ may be present. Furthermore, selected events must contain at least four jets. At least two of the jets in the event must be *b*-tagged. Finally, $E_{{\text {T}}}^{{\text {miss}}}$ must exceed 20 GeV.

### Background determination

After the event selection, a variety of potential background sources remain. Several sources that contain top quarks contribute to the background, with events that contain a single top quark being the dominant contribution. In addition, production of $t\bar{t}+X$ with *X* being either a *W*, *Z*, or Higgs boson is an irreducible background, which is, however, expected to be negligible. Events that contain either two electroweak bosons, or one electroweak boson in association with jets can be misidentified as signal. However, only the $W+\text {jets}$ component is expected to contribute significantly. Finally, multijet processes where either a semileptonic decay of a hadron is wrongly reconstructed as an isolated lepton or a jet is misidentified as a lepton enter the signal selection. This last category is collectively called the non-prompt (NP) and fake lepton background.

All backgrounds are modelled using MC simulation, with the exception of the NP and fake lepton background, which is estimated using the matrix method [72, 73]. A sample enriched in NP and fake leptons is obtained by loosening the requirements on the standard lepton selections defined in Sect. 4.1. The efficiency of these “loose” leptons to satisfy the standard criteria is then measured separately for prompt and NP or fake leptons. For both the electrons and muons the efficiency for a prompt loose lepton to satisfy the standard criteria is measured using a sample of *Z* boson decays. The efficiency for NP or fake loose electrons to satisfy the standard criteria is measured in events with low missing transverse momentum and the efficiency for NP or fake loose muons to pass the standard criteria is measured using muons with a high impact parameter significance. These efficiencies allow the number of NP and fake leptons selected in the signal region to be estimated.

The number of selected events is listed in Table 2. The estimated signal purity is approximately 88%, with the backgrounds from single top quarks and non-prompt and fake leptons being the largest impurities. In this analysis, the $t\bar{t}$ signal includes dilepton $t\bar{t}$ events in which one of the leptons is not identified. These events make up 9.8% of the total $t\bar{t}$ signal.

**Table 2:** Event yields after selection. The uncertainties include experimental uncertainties and the uncertainty in the data-driven non-prompt and fake lepton background. Theoretical cross-section uncertainties and uncertainties due to limited MC sample sizes are not included. Details of the uncertainties considered can be found in Sect. 7

Sample	Yield
$t\bar{t}$	$1026000 \pm 95000$
$t\bar{t}V$	$3270\pm 250$
$t\bar{t}H$	$1700\pm 100$
Single-top	$48400\pm 5500$
Diboson	$1440\pm 220$
$W+\text {jets}$	$27700\pm 4700$
$Z+\text {jets}$	$8300\pm 1400$
NP/Fake leptons	$53000\pm 30000$
Total expected	$1170000\pm 100000$
Observed	1153003

## Observable definition and reconstruction

The jet-pull vector is calculated from inner-detector tracks created using an updated reconstruction algorithm [74] that makes use of the newly introduced IBL [10] as well as a neural-network-based clustering algorithm [75, 76] to improve the pixel cluster position resolution and the efficiency of reconstructing tracks in jets. A measurement based on the calorimeter energy clusters of the jet is not considered in this analysis as it suffers from a significantly degraded spatial resolution, as was shown in Ref. [7].

To ensure good quality, reconstructed tracks must satisfy $\left| \eta \right| < 2.5$ and $p_{{\text {T}}} > 0.5\,\hbox {GeV}$, and further quality cuts are applied to ensure that they originate from and are assigned to the primary vertex [76].^4^ This suppresses contributions from pile-up and tracks with a poor quality fit that are reconstructed from more than one charged particle. Matching of tracks to jets is performed using a technique called ghost association [77], in which inner-detector tracks are included in the jet clustering procedure after having scaled their four-momenta to have infinitesimal magnitude. As a result, the tracks have no effect on the jet clustering result whilst being matched to the jet that most naturally encloses them according to the jet algorithm used. After the matching procedure, the original track four-momenta are restored. The jets used in calculating each observable are required to satisfy $|\eta |<2.1$ so that all associated tracks are within the coverage of the inner detector. Furthermore, at least two tracks must contribute to the pull-vector calculation.

The jet axis used to calculate the constituent offsets, $\vec {\Delta r}_{i}$, in Eq. (1) is calculated using the ghost-associated tracks, with their original four-momenta, rather than using the jet axis calculated from the calorimeter energy clusters that form the jet. This ensures proper correspondence between the pull vector and the constituents entering its calculation. For consistency, the total jet $p_{{\text {T}}}$ in Eq. (1) is also taken from the four-momentum of the recalculated jet axis.

The analysis presented in this paper measures the colour flow for two cases:

1. The signal colour flow is extracted from an explicitly colour-connected dijet system.
2. The spurious colour flow is obtained from a jet pair for which no specific colour connection is expected.

The study of the signal colour flow is performed using the candidate daughters of the hadronically decaying *W* boson from the top-quark decay. In practice, the two leading (highest-$p_{{\text {T}}}$) jets that have not been *b*-tagged are selected as *W* boson daughter candidates. A dedicated study using simulated $t\bar{t}$ events has shown that this procedure achieves correct matching of both jets in about 30% of all events, with roughly 50% of all cases having a correct match to one of the two jets. This reduces the sensitivity of this analysis to different colour model predictions compared with the ideal case of perfect identification of the *W* boson daughter jets. Nevertheless, the procedure is still sufficient to distinguish between the colour models considered in this analysis.

**Fig. 4:**
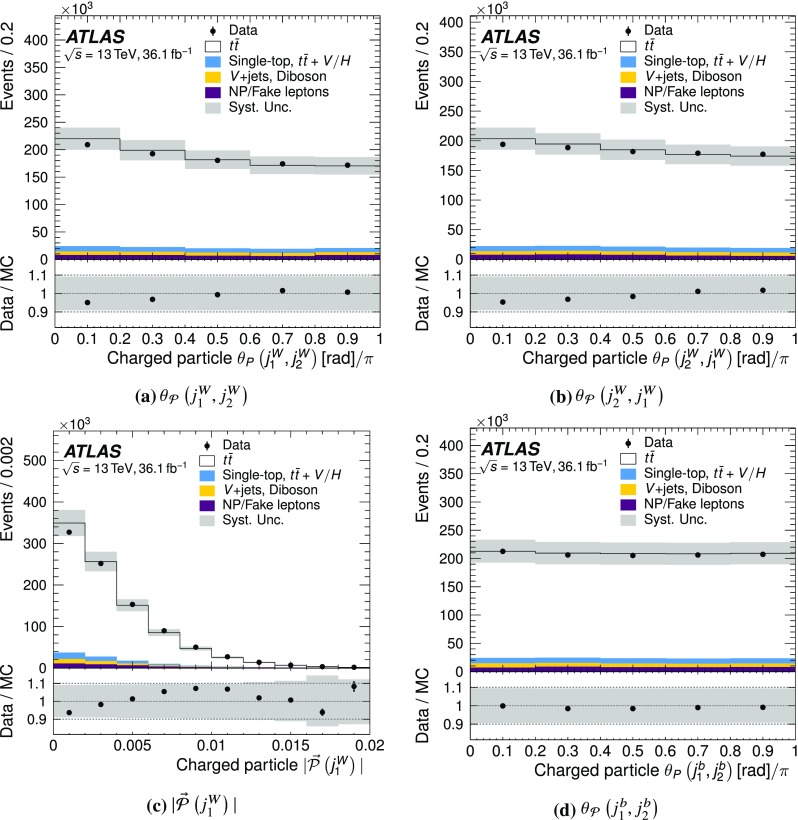
Detector-level distributions for the four considered observables: the **a** forward and **b** backward pull angle for the hadronically decaying *W* boson daughters, **c** the magnitude of the leading *W* daughter’s jet-pull vector, and **d** the forward di-*b*-jet-pull angle. Uncertainty bands shown include the experimental uncertainties and the uncertainty in the data-driven non-prompt and fake lepton background. Details of the uncertainties considered can be found in Sect. 7

The two jets assigned to the hadronically decaying *W* boson are labelled as $j_1^W$ and $j_2^W$, with the indices referring to their $p_{{\text {T}}}$ ordering. This allows the calculation of two jet-pull angles: $\theta _{\mathcal {P}}\left( j_1^W, j_2^W \right)$ and $\theta _{\mathcal {P}}\left( j_2^W, j_1^W \right)$, which are labelled as “forward pull angle” and “backward pull angle”, respectively. Although the two observables probe the same colour structure, in practice the two values obtained for a single event have a linear correlation of less than 1% in data and can be used for two practically independent measurements. Figure 4a, b compare the distributions observed for these two pull angles to those predicted by simulation at detector level.

In addition, the magnitude of the jet-pull vector is calculated for the jet with larger transverse momentum: $|\vec {\mathcal {P}} \left( j_1^W \right) |$. A comparison of the observed and predicted distributions for this observable can be found in Fig. 4(c), which shows a steeply falling distribution largely contained in the region below 0.005.

In $t\bar{t}$ events an obvious candidate for measuring spurious colour flow is the structure observed between the two leading *b*-tagged jets, as the partons that initiate the *b*-jets are not expected to have any specific colour connection. For a typical signal event, their colour charge can be traced to the gluon that splits into the $t\bar{t}$ pair. This coloured initial state ensures that the two *b*-quarks are not expected to be colour connected. Therefore, the forward di-*b*-jet-pull angle is calculated from the two leading *b*-tagged jets: $\theta _{\mathcal {P}}\left( j_1^b, j_2^b \right)$. According to the $t\bar{t}$ simulation, this choice achieves correct matching for both jets in about 80% of all events. Figure 4d shows a comparison of the distribution observed in data to that predicted by simulation for this observable. Consistent with the expectation, the distribution is flat, unlike in the case of the jet pairs from *W* boson decays.

Table 3 summarises the analysis observables and their definitions.

**Table 3:** Summary of the observables’ definitions

**Target colour flow**	**Signal colour flow**	**Spurious colour flow**
	($j_1$ and $j_2$ are colour connected)	($j_1$ and $j_2$ are not colour connected)
**Jet assignment**	$j_1^W$ : leading $p_{{\text {T}}}$ non-*b*-tagged jet	$j_1^b$ : leading $p_{{\text {T}}}$ *b*-tagged jet
	$j_2^W$ : $2{\text {nd}}$ leading $p_{{\text {T}}}$ non-*b*-tagged jet	$j_2^b$ : $2{\text {nd}}$ leading $p_{{\text {T}}}$ *b*-tagged jet
**Observables**	$\theta _{\mathcal {P}}\left( j_1^W, j_2^W \right)$ : *“forward pull-angle”*	$\theta _{\mathcal {P}}\left( j_1^b, j_2^b \right)$ : *“forward di-* *b* *-jet-pull angle”*
	$\theta _{\mathcal {P}}\left( j_2^W, j_1^W \right)$ : *“backward pull-angle”*	
	$|\vec {\mathcal {P}}\left( j_1^W \right) |$ : *“pull-vector magnitude”*	

## Unfolding

Particle-level objects are selected in simulated events using definitions analogous to those used at detector level, as discussed in the previous section. Particle-level objects are defined using particles with mean lifetime greater than $30\,\hbox {ps}$.

Electrons and muons must not originate from a hadron in the MC generator-level event record, either directly or through an intermediate $\tau$-lepton decay. In effect, this means that the lepton originates from a real *W* or *Z* boson. To take into account final-state photon radiation, the lepton four-momentum is modified by adding to it all photons not originating from a hadron that are within a $\Delta R = 0.1$ cone around the lepton. Leptons are then required to satisfy $p_{{\text {T}}} > 25\,\hbox {GeV}$ and $\left| \eta \right| < 2.5$.

Particle-level jets are constructed by clustering all stable particles, excluding leptons not from hadron decays and their radiated photons, using the same clustering algorithm and configuration as is used for the detector-level jets. Particle-level jets are furthermore required to satisfy $p_{{\text {T}}} > 25\,\hbox {GeV}$ and $\left| \eta \right| < 2.5$. Classification of jets as having originated from a *b*-hadron is performed using ghost association [77] where the *b*-hadrons considered for the procedure must satisfy $p_{{\text {T}}} > 5\,\hbox {GeV}$. This is equivalent to the method used for matching tracks to jets described in Sect. 5, except that it is applied during particle-level jet clustering and adds ghosts for unstable *b*-hadrons rather than inner-detector tracks. A particle-level jet is considered to be *b*-tagged if it contains at least one such *b*-hadron.

An overlap removal procedure is applied that rejects leptons that overlap geometrically with a jet at $\Delta R < 0.4$.

The magnitude of the missing transverse momentum $E_{{\text {T}}}^{{\text {miss}}}$ at particle level is calculated as the transverse component of the four-momentum sum of all neutrinos in the event excluding those from hadron decays, either directly or through an intermediate $\tau$-lepton decay.

At particle level, the event selection requires exactly one lepton with $p_{{\text {T}}} > 27\,\hbox {GeV}$ with no additional lepton, at least four jets of which at least two are *b*-tagged, as well as $E_{{\text {T}}}^{{\text {miss}}} > 20\,\hbox {GeV}$.

At particle level, the input to the calculation of the jet-pull vector is the collection of jet constituents as defined by the clustering procedure described in Sect. 4.1. To reflect the fact that the detector-level observable’s definition uses tracks, only charged particles are considered. Furthermore, a requirement of $p_{{\text {T}}} > 0.5\,\hbox {GeV}$ is imposed in line with the detector-level definition to reduce simulation-based extrapolation and associated uncertainties. Apart from the inputs to the jet-pull-vector calculation, the procedure applied at detector level is mirrored exactly at particle level.

The measured distributions are unfolded using the iterative Bayesian method [78] as implemented by the RooUnfold framework [79]. This algorithm iteratively corrects the observed data to an unfolded particle-level distribution given a certain particle-level prior. Initially, this prior is taken to be the particle-level distribution obtained from simulation. However, it is updated after each iteration with the observed posterior distribution. Thus, the algorithm converges to an unfolded result driven by the observed distribution. The number of iterations used by the unfolding method is chosen such that the total uncertainty composed of the statistical uncertainty and the bias is minimised.

The measurement procedure consists essentially of two stages: first the background contributions are subtracted bin-by-bin from the observed data. Secondly, detector effects are unfolded from the signal distribution using a detector response model, the migration matrix, obtained from simulated $t\bar{t}$ events. As part of this second step, two correction factors are applied that correct for non-overlap of the fiducial phase space at detector- and particle-level. The corrections account for events that fall within the fiducial phase space of one level but not the other. The full procedure for an observable *X* can be summarised symbolically by the equation

$$\begin{aligned} \frac{\mathrm {d} \sigma _{\text {Fid}}^{t}}{\mathrm {d} X^{t}} = \frac{1}{\mathcal {L} \cdot \Delta X^{t}} \cdot \frac{1}{\epsilon ^t} \sum _{r} \mathcal {M}_{r,t}^{-1} \cdot \epsilon _{\text {Fid}}^{r} \cdot \left( N_{\text {Obs}}^{r} - N_{{\text {Bkg}}}^{r} \right) , \end{aligned}$$

where *t* indicates the particle-level bin index, *r* the detector-level bin index, $\mathcal {L}$ is the integrated luminosity of the data, $\mathcal {M}_{r,t}$ is the migration matrix and the inversion symbolises unfolding using the iterative Bayesian method, $N_{{\text {Obs}}}^{r}$ is the number of observed events, $N_{{\text {Bkg}}}^{r}$ the expected number of background events, and $\epsilon ^t$ and $\epsilon _{{\text {Fid}}}^r$ are the phase-space correction factors. These last two parameters are defined as

$$\begin{aligned} \epsilon ^t = \frac{N_{{\text {PL}}\wedge \text {RL}}}{N_{{\text {PL}}}} \quad \epsilon ^r_{{\text {Fid}}} = \frac{N_{{\text {PL}}\wedge {\text {RL}}}}{N_{{\text {RL}}}}. \end{aligned}$$

The number $N_{{\text {PL}}}$ ($N_{{\text {RL}}}$) indicates the number of events fulfilling the fiducial requirements at particle level (selection requirements at detector level), $N_{{\text {PL}}\wedge {\text {RL}}}$ is the number of events that pass both sets of requirements at their respective level.

The response model and phase-space correction factors are obtained from $t\bar{t}$ simulation. The values of $\epsilon ^r_{{\mathrm {Fid}}}$ are reasonably independent of the variable for all variables considered, and are $\approx 70\%$. The values of $\epsilon ^t$ are also reasonably independent of the variable for the three pull angles, while for the pull-vector magnitude, $\epsilon ^t$ varies from $\approx 72\%$ at small values to $\approx 67\%$ at higher values.

Some of the background samples considered in this analysis potentially contain true signal colour flow, e.g. the single-top or $t\bar{t}+X$ contributions. However, as their overall contributions are very small, even extreme changes in their respective colour flow have a negligible effect. Therefore, all such contributions are ignored and the estimated backgrounds, with SM colour flow assumed, are subtracted from the data.

The binning chosen for the observables is determined by optimisation studies performed with simulated samples. A good binning choice should result in a mostly diagonal migration matrix with bin widths appropriate to the observed resolution. The optimisation therefore imposes a requirement of having at least 50% of events on-diagonal for each particle-level bin of the migration matrix. The resulting migration matrices typically have $> 55\%$ of events on-diagonal.

## Treatment of uncertainties

Several systematic uncertainties affect the measurements discussed above. The different sources are grouped into four categories: experimental uncertainties, uncertainties related to the modelling of the signal process, uncertainties related to the modelling of the background predictions, and an uncertainty related to the unfolding procedure.

The changes that result from variations accounting for sources of systematic uncertainty are used to calculate a covariance matrix for each source individually. This covariance matrix combines the changes from all measured observables simultaneously, and therefore also includes the cross-correlations between observables. The total covariance matrix is then calculated by summation over the covariances obtained from all sources of systematic uncertainty. The changes observed for a source of systematic uncertainty are symmetrised prior to calculating the covariance. For one-sided variations, the change is taken as a symmetric uncertainty. For two-sided variations, which variation is used to infer the sign is completely arbitrary, as long as it is done consistently. In this analysis, the sign – which is only relevant for the off-diagonal elements of the covariance matrix – is taken from the upward variation while the value is taken as the larger change. Furthermore, it is assumed that all uncertainties, including modelling uncertainties, are Gaussian-distributed.

### Experimental uncertainties

Systematic uncertainties due to the modelling of the detector response and other experimental sources affect the signal reconstruction efficiency, the unfolding procedure, and the background estimate. Each source of experimental uncertainty is treated individually by repeating the full unfolding procedure using as input a detector response that has been varied appropriately. The unfolding result is then compared to the nominal result and the difference is taken as the systematic uncertainty. Through this procedure the measured data enter the calculation for each source of experimental uncertainty.

Uncertainties due to lepton identification, isolation, reconstruction, and trigger requirements are evaluated by varying the scale factors applied in the simulation to efficiencies and kinematic calibrations within their uncertainties. The scale factors and an estimate of their uncertainty were derived from data in control regions enriched in $Z \rightarrow \ell \ell , W \rightarrow \ell \nu$, or $J/\psi$ events  [60, 80–82].

The uncertainties due to the jet energy scale (JES) and resolution (JER) are derived using a combination of simulation, test-beam data, and *in situ* measurements [65, 83–86]. In addition, contributions from $\eta$-intercalibration, single-particle response, pile-up, jet flavour composition, punch-through, and varying calorimeter response to different jet flavours are taken into account. This results in a scheme with variations for 20 systematic uncertainty contributions to the JES.

Efficiencies related to the performance of the *b*-tagging procedure are corrected in simulation to account for differences between data and simulation. The corresponding scale factors are extracted from simulated $t\bar{t}$ events. This is done separately for *b*-jets, *c*-jets, and light jets, thereby accounting for mis-tags. Uncertainties related to this procedure are propagated by varying the scale factors within their uncertainty [68, 87, 88].

The uncertainties on the $E_{{\text {T}}}^{{\text {miss}}}$ due to systematic shifts in the corrections for leptons and jets are accounted for in a fully correlated way in their evaluation for those physics objects. Uncertainties due to track-based terms in the $E_{{\text {T}}}^{{\text {miss}}}$ calculation, i.e. those that are not associated with any other reconstructed object, are treated separately [89].

All uncertainties associated with the reconstructed tracks directly enter the observable calculation as defined in Eq. (1). Uncertainties are either expressed as a change in the tracking efficiency or smearing of the track momentum [74, 76]. This also includes effects due to fake tracks and lost tracks in the core of jets. Corrections and scale factors were extracted using simulated data as well as experimental data obtained from minimum-bias, dijet, and $Z \rightarrow \mu \mu$ selections. The systematic shifts applied as part of this procedure are in most cases parameterised as functions of the track $p_{{\text {T}}}$ and $\eta$, see Ref. [74].

The uncertainty in the combined 2015 and 2016 integrated luminosity is 2.1%, which is derived following a method similar to that detailed in Ref. [90], from a calibration of the luminosity scale using *x*–*y* beam-separation scans performed in August 2015 and May 2016. This uncertainty affects the scaling of the background prediction that is subtracted from the observed data. The uncertainty related to the pile-up reweighting is evaluated by varying the scale factors by their uncertainty based on the reweighting of the average number of interactions per *pp* collision.

The data’s statistical uncertainty and bin-to-bin correlations are evaluated using the bootstrap method [91]. Bootstrap replicas of the measured data are propagated through the unfolding procedure and their variance is used to assess the statistical uncertainty. These replicas can also be used to calculate the statistical component of the covariance of the measurement as well as the statistical bin-by-bin correlations of the pre- or post-unfolding distributions.

### Signal modelling uncertainties

The following systematic uncertainties related to the modelling of the $t\bar{t}$ system are considered: the choice of matrix-element generator, the choice of PDF, the hadronisation model, the amount of initial- and final-state radiation (ISR/FSR), and the amount and strength of colour reconnection (CR).

Signal modelling uncertainties are evaluated individually using different signal MC samples. Detector-level distributions from the alternative signal MC sample are unfolded using the nominal response model. The unfolding result is then compared to the particle-level prediction of the alternative MC sample and the difference is used as the uncertainty. Table 1 lists the alternative signal MC samples used for assessing the generator, hadronisation, ISR/FSR systematic uncertainties (A14.v3c tune variations), and CR (A14.v1 tune variations) systematic uncertainties.

The uncertainty arising from the choice of PDF is evaluated by creating reweighted pseudo-samples, in which the weight variations for the PDF sets are according to the PDF4LHC [92] prescription. The unfolding results obtained for the pseudo-samples are then combined in accordance with the PDF4LHC procedure to obtain a single systematic shift.

### Background modelling uncertainties

Systematic uncertainties related to the background modelling affect the number of background events subtracted from data prior to the unfolding.

The normalisation of the background contributions obtained from MC simulation is varied within the uncertainties obtained from the corresponding cross-section calculation. For the single-top background, the normalisation uncertainty ranges from 3.6 to 5.3% [41–43], and for the $t\bar{t}Z$ and $t\bar{t}W$ backgrounds it is 12% and 13%, respectively [46, 47]. In the case of the $W/Z+\text {jets}$ backgrounds, the uncertainties include a contribution from the overall cross-section normalisation (4%), as well as an additional 24% uncertainty added in quadrature for each jet [93, 94]. For the diboson background, the normalisation uncertainty is 6% [95]. The uncertainty of the normalisation for the $t\bar{t}H$ background is chosen to be 100%.

The uncertainty arising from the modelling of the non-prompt and fake lepton background is assessed by varying the normalisation by 50%, as well as by changing the efficiency parameterisation used by the matrix method [72, 73] to obtain a shape uncertainty. These uncertainties were found to cover adequately any disagreement between data and prediction in various background-dominated control regions.

The uncertainty due to the level of radiation in the single-top background is evaluated using two alternative simulation samples with varied levels of radiation. These two samples were generated using the same approach that was used to produce the radiation variation samples of the nominal $t\bar{t}$ process. At NLO QCD the *tW*-channel single-top process, which contributes around 70% of the total single-top background in this analysis, and the $t\bar{t}$ process can share the same final state and therefore interfere. The uncertainty due to this higher-order overlap between the $t\bar{t}$ and *tW* processes is evaluated by assessing the impact of replacing the nominal *tW* MC sample, which accounts for overlap using the “diagram removal” scheme, with an alternative *tW* MC sample that accounts for the overlap using the “diagram subtraction” scheme [31].

A *tW* colour-model uncertainty is considered, which is motivated by the overlap between the $t\bar{t}$ and *tW* processes. This overlap implies that the colour flow in *tW* is of the same type as the signal colour flow in the $t\bar{t}$ process. However, the *tW* colour flow is estimated from simulation and subtracted from data prior to unfolding. Hence, mismodelling of the *tW* colour flow would affect the unfolding result. An uncertainty is constructed by reweighting the combination of $t\bar{t}$ and *tW* to have the same shape as data. For evaluation of the systematic uncertainty, the reweighted *tW* is then considered for the background subtraction and unfolding is repeated.

### Unfolding procedure systematic uncertainty

The uncertainty arising from the unfolding procedure, also called the non-closure uncertainty, is assessed using a data-driven approach. For each measured distribution, simulated particle-level events are reweighted using a linear weight function such that the corresponding detector-level distributions are in better agreement with the data. The weights are propagated to the corresponding detector-level events and the resulting reweighted distributions are unfolded using the nominal detector-response model. Deviations of these unfolded distributions from the reweighted particle-level distributions are then assigned as the non-closure uncertainty.

A summary of the uncertainties affecting $\theta _{\mathcal {P}}\left( j_1^W, j_2^W \right)$ is shown in Table 4. The total uncertainty is dominated by systematic uncertainties, with those accounting for $t\bar{t}$ modelling being dominant in most bins. Uncertainties that directly affect the inputs to the pull-vector calculation, such as the JES, JER and track uncertainties are generally sub-dominant.

The systematic uncertainties in Table 4 are much smaller than those shown in Table 2 and Fig. 4. This is because Table 4 gives the uncertainties appropriate for a comparison between normalised distributions in which overall scale uncertainties play no role. As a result, many of the experimental uncertainties, which have little to no impact on the shape of the measured distributions, also have a reduced effect on the measurement. For example, the uncertainties due to *b*-tagging reduce from around 7.5% to less than 0.5%.

**Table 4:** Statistical and systematic uncertainties affecting the measurement of $\theta _{\mathcal {P}} \left( j_1^W, j_2^W \right)$. The category “Other” summarises various smaller uncertainty components. Uncertainties are ordered by the mean value of the uncertainty across all bins and are expressed in percent of the measured value

$\Delta \theta _P\left( j_{1}^{W}, j_{2}^{W} \right) \, [\%]$	$\theta _P\left( j_{1}^{W}, j_{2}^{W} \right)$			
	$0.0{-}0.21$	$0.21{-}0.48$	$0.48-0.78$	$0.78{-}1.0$
Hadronisation	0.55	0.13	0.24	0.14
Generator	0.32	0.25	0.50	0.01
*b*-tagging	0.35	0.13	0.20	0.31
Background model	0.30	0.16	0.16	0.27
Colour reconnection	0.22	0.16	0.16	0.18
JER	0.11	0.12	0.23	0.02
Pile-up	0.19	0.16	0.00	0.01
Non-closure	0.14	0.07	0.07	0.18
JES	0.12	0.06	0.14	0.06
ISR / FSR	0.15	0.02	0.12	0.02
Tracks	0.05	0.04	0.03	0.06
Other	0.02	0.01	0.01	0.02
Syst.	0.88	0.44	0.71	0.51
Stat.	0.23	0.19	0.19	0.25
Total	0.91	0.48	0.73	0.57

## Results

**Fig. 5:**
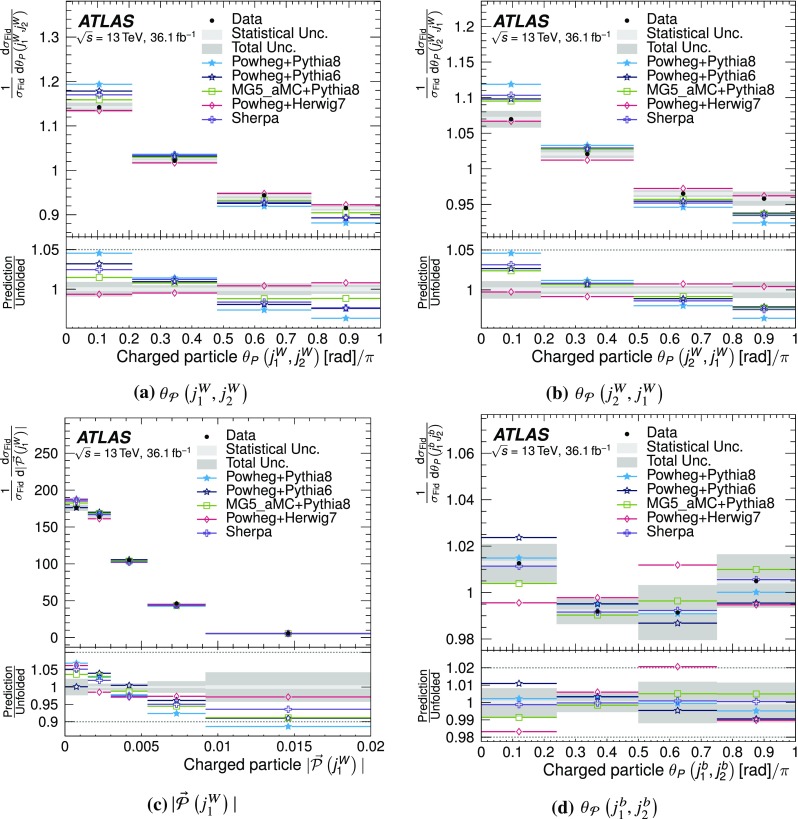
Normalised fiducial differential cross-sections as a function of the **a** forward and **b** backward pull angle for the hadronically decaying *W* boson daughters, **c** the magnitude of the leading *W* daughter’s jet-pull vector, and **d** the forward di-*b*-jet-pull angle. The data are compared to various SM predictions. The statistical uncertainties in the predictions are smaller than the marker size

Figure 5 compares the normalised unfolded data to several Standard Model (SM) predictions, summarised in Table 1, for all four observables. Three SM predictions use Powheg to generate the hard-scatter events and then differ for the subsequent hadronisation, namely Pythia 6, Pythia 8, and Herwig 7. A main difference between these predictions is that the Pythia family uses the colour string model [96] while Herwig uses the cluster model [20] for hadronisation. One SM prediction uses MG5_aMC to produce the hard-scatter event, the hadronisation is then performed using Pythia 8. Finally, one SM prediction is obtained from events generated with Sherpa.

**Fig. 6:**
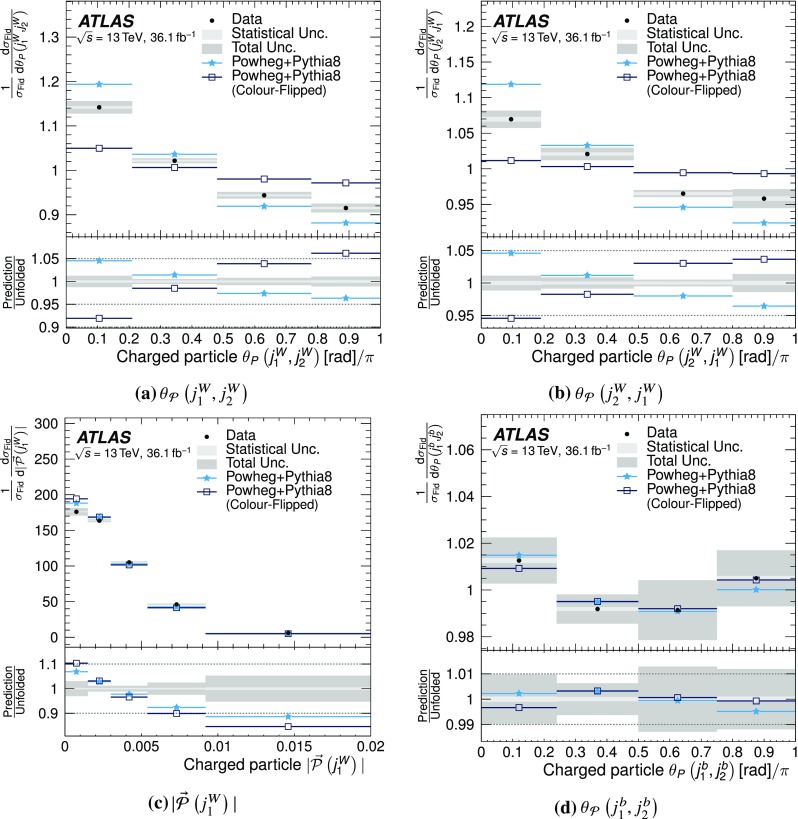
Normalised fiducial differential cross-sections as a function of the **a** forward and **b** backward pull angle for the hadronically decaying *W* boson daughters, **c** the magnitude of the leading *W* daughter’s jet-pull vector, and **d** the forward di-*b*-jet-pull angle. The data are compared to a SM prediction produced with Powheg + Pythia 8 as well as the model with exotic colour flow also created with Powheg + Pythia 8. The uncertainty bands presented in these plots combine the baseline set of systematic uncertainties with effects due to considering the exotic colour-flipped model as a source of signal modelling uncertainty. The statistical uncertainties in the predictions are smaller than the marker size

Figure 6 compares the normalised unfolded data to the SM prediction as well as a prediction obtained from the exotic model with flipped colour flow described in Sect. 3. Both predictions are obtained from MC samples generated with Powheg + Pythia 8. The data agree better with the SM prediction than the colour-flipped sample.

The uncertainty bands on the unfolding results shown in Fig. 6 include an additional “colour model uncertainty”. This uncertainty is obtained using the same procedure that is used for the signal modelling uncertainties, using the sample with exotic colour flow as the alternative $t\bar{t}$ MC sample. It has a similar size to the dominant signal-modelling uncertainties.

A goodness-of-fit procedure is employed in order to quantify the level of agreement between the measured distributions and those predicted by the MC generators. A $\chi ^2$ test statistic is calculated for each pairing of an observable and the theoretical prediction individually, using the full covariance matrix of the experimental uncertainties, but excluding any uncertainties in the theoretical predictions. Given the unfolded data *D*, the model prediction *M*, and the covariance $\Sigma$, the $\chi ^2$ is given by

$$\begin{aligned} \chi ^2 = (D^T - M^T) \cdot \Sigma ^{-1} \cdot (D - M). \end{aligned}$$

Subsequently, *p*-values can be calculated from the $\chi ^2$ and number of degrees of freedom (NDF), and these are the probability to obtain a $\chi ^2$ value greater than or equal to the observed value.

The fact that the analysis measures normalised distributions removes one degree of freedom from the $\chi ^2$ calculation. Consequently, one of the *N* elements of *D* and *M* is dropped and the covariance is reduced from dimensionality $N \times N$ to $(N-1) \times (N-1)$ by discarding one column and row. The $\chi ^2$ value does not depend on the choice of discarded elements. Table 5 lists the resulting $\chi ^2$ values and derived *p*-values.

**Table 5:** The $\chi ^2$ and resulting *p* values for the measured normalised cross-sections obtained by comparing the different predictions to the unfolded data. When comparing the data with the prediction for the exotic flipped colour-flow model, the model itself is considered as an additional source of signal modelling uncertainty and thus added to the covariance matrix. Calculations that include this additional systematic uncertainty are marked with $\star$

Sample	$\theta _{\mathcal {P}} \left( j_1^W, j_2^W \right)$		$\theta _{\mathcal {P}} \left( j_2^W, j_1^W \right)$		$\theta _{\mathcal {P}} \left( j_1^b, j_2^b \right)$		$|\vec {\mathcal {P}}\left( j_1^W \right) |$	
	$\chi ^2 / \text {NDF}$	*p*-value	$\chi ^2 / \text {NDF}$	*p* value	$\chi ^2 / \text {NDF}$	*p*-value	$\chi ^2 / \text {NDF}$	*p* value
Powheg + Pythia8	50.9 / 3	$< 0.001$	25.1 / 3	$< 0.001$	0.7 / 3	0.867	24.8 / 4	$< 0.001$
Powheg + Pythia6	23.2 / 3	$< 0.001$	8.2 / 3	0.042	4.2 / 3	0.240	21.1 / 4	$< 0.001$
MG5_aMC + Pythia8	6.8 / 3	0.077	6.7 / 3	0.082	2.0 / 3	0.563	17.6 / 4	0.001
Powheg + Herwig7	2.7 / 3	0.446	3.4 / 3	0.328	4.8 / 3	0.190	11.3 / 4	0.023
Sherpa	22.0 / 3	$< 0.001$	11.9 / 3	0.008	0.0 / 3	0.998	14.1 / 4	0.007
Powheg + Pythia8$^{\star }$	17.1 / 3	$< 0.001$	25.0 / 3	$< 0.001$	0.3 / 3	0.958	11.1 / 4	0.026
Flipped Powheg + Pythia8$^{\star }$	45.3 / 3	$< 0.001$	45.9 / 3	$< 0.001$	2.6 / 3	0.457	17.2 / 4	0.002

For the signal jet-pull angles $\theta _{\mathcal {P}}\left( j_1^W, j_2^W \right)$ and $\theta _{\mathcal {P}}\left( j_2^W, j_1^W \right)$, the predictions obtained from Powheg + Herwig 7 agree best with the observed data. A general trend is that simulation predicts a steeper distribution, i.e. a stronger colour-flow effect. The magnitude of the jet-pull vector is poorly modelled in general, with the prediction obtained from Powheg + Herwig 7 agreeing best with data. As with the signal jet-pull angles, the disagreement shows a similar trend for the different MC predictions: data favours larger values of the jet-pull vector’s magnitude. Predictions from Powheg + Pythia 6 are in significantly better agreement with the data than those obtained from Powheg + Pythia 8 for the signal jet-pull angles and jet-pull vector’s magnitude.

The signal jet-pull angles and the jet-pull vector’s magnitude can be used to distinguish the case of colour flow like that in the SM from the exotic flipped colour-flow scenario constructed in Sect. 3. The data favour the SM prediction over the colour-flipped prediction.

The forward di-*b*-jet-pull angle is modelled relatively well by most predictions. In particular the distribution obtained from Sherpa agrees extremely well with the measurement. Powheg + Herwig 7, which otherwise shows relatively good agreement with data for the other three observables, agrees least well of the tested predictions. Indeed, it is the only prediction that is consistently outside of the estimated uncertainty bands. As expected, the forward di-*b*-jet-pull angle $\theta _{\mathcal {P}}\left( j_1^b, j_2^b\right)$ does not show the sloped distribution that the signal jet-pull angles $\theta _{\mathcal {P}}\left( j_1^W, j_2^W\right)$ and $\theta _{\mathcal {P}}\left( j_2^W, j_1^W\right)$ follow.

## Conclusion

A measurement of four observables sensitive to the colour flow in $t\bar{t}$ events is presented, using $36.1\,\text {fb}^{-1}$ of $\sqrt{s} = 13\,\hbox {TeV}\,pp$ collision data recorded by the ATLAS detector at the LHC. The four observables are the forward and backward jet-pull angles for the *W* boson daughters, the magnitude of the jet-pull vector of the leading *W* boson daughter, and the jet-pull angle between the *b*-tagged jets. The measured distributions are compared to several theoretical predictions obtained from MC simulation.

The default SM prediction, Powheg + Pythia 8, agrees poorly with the data. However, alternative SM predictions exhibit much better agreement. In particular, the prediction obtained by Powheg + Herwig 7 provides a rather good description of the data. Predictions from Powheg + Pythia 6 are in significantly better agreement with the data than those obtained from Powheg + Pythia 8.

In addition, a model with exotic colour flow is compared to the data. In the observables sensitive to the exotic colour flow, data favours the SM case over the exotic model.

## Footnotes

1 ATLAS uses a right-handed coordinate system with its origin at the nominal interaction point (IP) in the centre of the detector and the *z*-axis along the beam pipe. The *x*-axis points from the IP to the centre of the LHC ring, and the *y*-axis points upward. Cylindrical coordinates $(r,\phi )$ are used in the transverse plane, $\phi$ being the azimuthal angle around the *z*-axis. The rapidity, which is used in the jet-pull vector calculation, is defined as $y = \frac{1}{2} \ln \frac{E + p_z}{E - p_z}$ using an object’s energy *E* and momentum $p_z$ along the *z*-axis. A related quantity is the pseudorapidity, which is defined in terms of the polar angle $\theta$ as $\eta =-\ln \tan (\theta /2)$. Using these coordinates, the radial distance $\Delta R$ between two objects is thus defined as $\Delta R = \sqrt{(\Delta \eta )^2 + (\Delta \phi )^2}$ where $\Delta \eta$ and $\Delta \phi$ are the differences in pseudorapidity and azimuthal angle between the two objects, respectively.

2 The term *tune* refers to a specific setting of configurable parameters of the MC generator describing non-perturbative QCD effects. A tune variation can be used to assess the effect of the modelling of non-perturbative effects on an analysis.

3 Electrons and muons produced via an intermediate $\tau$-lepton decay are also accepted.

4 Similar to the quality requirements used for the electron and muon reconstruction, cuts are applied such that the tracks satisfy $|d_0| < 2\,\hbox {mm}$ and $|z_0\cdot \sin \theta | < 3\,\hbox {mm}$.
